# Refining Established Practices for Research Question Definition to Foster Interdisciplinary Research Skills in a Digital Age: Consensus Study With Nominal Group Technique

**DOI:** 10.2196/56369

**Published:** 2025-01-23

**Authors:** Jana Sedlakova, Mina Stanikić, Felix Gille, Jürgen Bernard, Andrea B Horn, Markus Wolf, Christina Haag, Joel Floris, Gabriela Morgenshtern, Gerold Schneider, Aleksandra Zumbrunn Wojczyńska, Corine Mouton Dorey, Dominik Alois Ettlin, Daniel Gero, Thomas Friemel, Ziyuan Lu, Kimon Papadopoulos, Sonja Schläpfer, Ning Wang, Viktor von Wyl

**Affiliations:** 1 Digital Society Initiative University of Zurich Zurich Switzerland; 2 Institute of Implementation Science in Healthcare Faculty of Medicine University of Zurich Zurich Switzerland; 3 Epidemiology, Biostatistics and Prevention Institute Faculty of Medicine University of Zurich Zurich Switzerland; 4 Department of Informatics Faculty of Business, Economics and Informatics University of Zurich Zurich Switzerland; 5 Center for Gerontology University of Zurich Zurich Switzerland; 6 Department of Psychology Faculty of Arts and Social Sciences University of Zurich Zurich Switzerland; 7 Institute of Evolutionary Medicine Faculty of Medicine University of Zurich Zurich Switzerland; 8 Department of Computational Linguistics Faculty of Business, Economics and Informatics University of Zurich Zurich Switzerland; 9 Center of Dental Medicine Faculty of Medicine University of Zurich Zurich Switzerland; 10 Institute of Biomedical Ethics and History of Medicine Faculty of Medicine University of Zurich Zurich Switzerland; 11 Department of Surgery and Transplantation University Hospital of Zurich Zurich Switzerland; 12 Department of Communication and Media Research Faculty of Arts and Social Sciences University of Zurich Zurich Switzerland; 13 Institute of Implementation Science in Healthcare Faculy of Medicine University of Zurich Zurich Switzerland; 14 Institute for Complementary and Integrative Medicine University Hospital of Zurich Zurich Switzerland

**Keywords:** research question, digitalization, digital data, data science, health research, interdisciplinary

## Abstract

**Background:**

The increased use of digital data in health research demands interdisciplinary collaborations to address its methodological complexities and challenges. This often entails merging the linear deductive approach of health research with the explorative iterative approach of data science. However, there is a lack of structured teaching courses and guidance on how to effectively and constructively bridge different disciplines and research approaches.

**Objective:**

This study aimed to provide a set of tools and recommendations designed to facilitate interdisciplinary education and collaboration. Target groups are lecturers who can use these tools to design interdisciplinary courses, supervisors who guide PhD and master’s students in their interdisciplinary projects, and principal investigators who design and organize workshops to initiate and guide interdisciplinary projects.

**Methods:**

Our study was conducted in 3 steps: (1) developing a common terminology, (2) identifying established workflows for research question formulation, and (3) examining adaptations of existing study workflows combining methods from health research and data science. We also formulated recommendations for a pragmatic implementation of our findings. We conducted a literature search and organized 3 interdisciplinary expert workshops with researchers at the University of Zurich. For the workshops and the subsequent manuscript writing process, we adopted a consensus study methodology.

**Results:**

We developed a set of tools to facilitate interdisciplinary education and collaboration. These tools focused on 2 key dimensions— content and curriculum and methods and teaching style—and can be applied in various educational and research settings. We developed a glossary to establish a shared understanding of common terminologies and concepts. We delineated the established study workflow for research question formulation, emphasizing the “what” and the “how,” while summarizing the necessary tools to facilitate the process. We propose 3 clusters of contextual and methodological adaptations to this workflow to better integrate data science practices: (1) acknowledging real-life constraints and limitations in research scope; (2) allowing more iterative, data-driven approaches to research question formulation; and (3) strengthening research quality through reproducibility principles and adherence to the findable, accessible, interoperable, and reusable (FAIR) data principles.

**Conclusions:**

Research question formulation remains a relevant and useful research step in projects using digital data. We recommend initiating new interdisciplinary collaborations by establishing terminologies as well as using the concepts of research tasks to foster a shared understanding. Our tools and recommendations can support academic educators in training health professionals and researchers for interdisciplinary digital health projects.

## Introduction

### Background

Health research increasingly leverages the abundance of data from our “digital lives,” including mobility data, social media data, or data from wearables [1,2]. Such digital data are commonly “unstructured” because it may not conform to a tabular format (eg, images, videos, sound, and free text) and often require specific expertise for harvesting; transforming; preprocessing; and creating meaningful insights into health, disease, and treatment [1,3-5]. Moreover, such digital data are often originally generated for nonresearch purposes and without addressing a specific research question [6]. In turn, they may lack standard quality attributes found in digital data collected for specific research purposes, such as depth, completeness, or consistency, which present methodological complexities to meaningfully use these data [1]. Therefore, reusing these digital unstructured data for health research requires diverse expertise, skills, and interdisciplinary collaboration between health domain experts (eg, clinicians and health scientists) and data scientists as method experts (eg, from data science, computer science, or statistics) [5,7,8].

Such interdisciplinary collaborations are often faced with challenges due to the seemingly conflicting research approaches between the disciplines. In addition to differences in terminologies and concept definitions, the prevailing emphasis of linear deductive approaches in health research contrasts with the often more explorative and iterative approaches used in data science [7]. In health research, it is customary to predefine key elements of the scientific process, including a research question and related hypothesis, in a protocol and scientific report (eg, STROBE [Strengthening the Reporting of Observational Studies in Epidemiology] or PRISMA [Preferred Reporting Items for Systematic Reviews and Meta-Analyses] guidelines) [9-12]. These standard practices are deeply influenced by the tradition of clinical trials and treatment development, which place a strong emphasis on measurement validity, robustness, scientific rigor, and safety [13], as errors in study conduct or treatment could place study participants at risk. By contrast, data science generally tends to emphasize exploration, pattern discovery, or hypothesis generation as well as more iterative and inductive analysis approaches [1,14]. Some health researchers may perceive this greater emphasis on iterative approaches as lacking scientific rigor or focus on specific research questions.

For young researchers, interdisciplinary digital health collaborations might be particularly challenging because they need to balance traditional scientific methods with more iterative data-driven techniques. This dual demand highlights the importance of fostering interdisciplinary skills in education, enabling students to balance the rigorous demands of hypothesis-driven research with the iterative and inductive approaches of data science. Addressing these complexities represents an educational challenge for both established and young researchers.

Despite broad recognition of their importance, both practical and teaching or educational guidance on how to manage and overcome the challenges of interdisciplinary digital health collaborations are scarce. Such guidance is also important for educational purposes to foster skills for interdisciplinary collaboration among both young and established researchers as well as health professionals. Continuous education for experienced researchers is equally important to keep them updated with evolving methods and foster effective collaboration across disciplines.

### This Study

To address this need, our study focuses on skill development to successfully navigate interdisciplinary collaborations and education in health-related research fields. We reviewed established workflows for research question formulation and investigated whether and how established workflows in health research may require adaptations to accommodate inductive and exploratory data science practices and novel analysis techniques. The study findings were translated into a set of tools and recommendations designed to facilitate interdisciplinary education and collaboration. These tools focus on 2 key dimensions—*content and curriculum* and *methods and teaching style*—and can be applied in various educational and research settings. Lecturers can use them to design interdisciplinary courses, supervisors can guide PhD and master’s students in their interdisciplinary projects, and principal investigators can design and organize workshops to initiate and guide interdisciplinary projects. By implementing these tools, educators and researchers can create more cohesive and productive educational resources for interdisciplinary collaborations. In the following sections, we offer our insights and a more detailed outline of how our study findings can inform both the *content* and *methods* dimensions, using an existing interdisciplinary course as an example.

The aims and findings of our study are intended to be globally relevant and applicable to all researchers using digital data in the context of health research and health care. Importantly, they also provide academic educators with a clear workflow and practical recommendations for discussing and addressing the challenges of interdisciplinary collaboration. As the focus is on research question formulation, a fundamental aspect of the research process, these recommendations are especially valuable for educational purposes, helping educators guide researchers and students through this essential phase of research projects.

To achieve our aims, we chose a consensus study approach that is appropriate to harmonize and bridge insights from experts from diverse research disciplines. Moreover, we focused our effort on the different approaches of research question formulation as the guiding example for this study because it represents a central step in guiding the research process and subsequent study design decisions. This process also served as an illustrative example to highlight the differences in research approaches between health research and data science.

## Methods

### Consensus Methods

We used the nominal group technique with expert groups to gather insights from a diverse range of experts. This approach aimed to foster interdisciplinary skills and knowledge and achieve consensus on adapting research question development.

**Figure 1:**
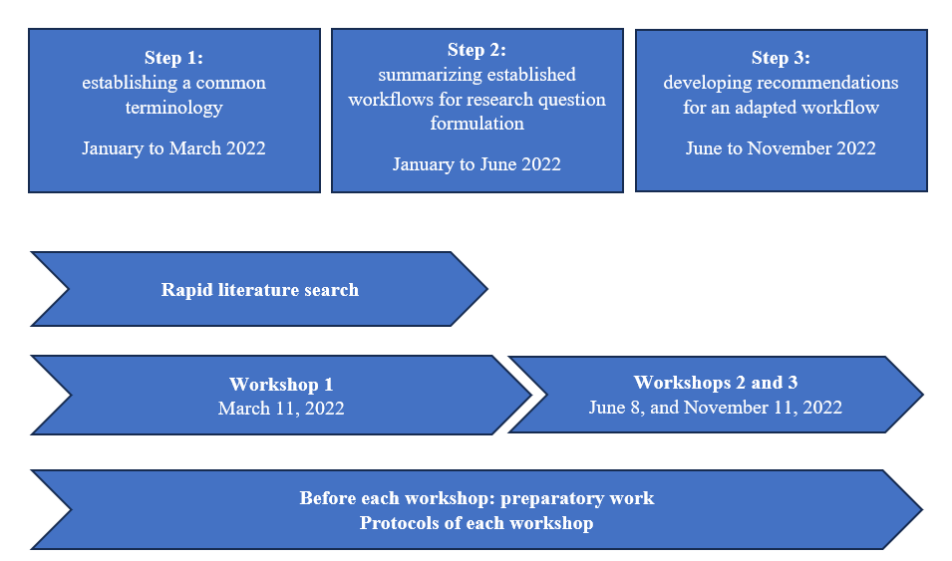
Study flow.

This study was structured by the following three high-level steps (Figure 1):

1. To create a common terminology to facilitate interdisciplinary and transdisciplinary collaborations that are required for research projects reusing digital data (ie, repurposing data originally generated for nonresearch purposes)
2. To describe the “established workflow” for research question formulation in health research on the basis of existing literature
3. To formulate suggestions and recommendations for adapting the “established workflow”

To inform steps 1 and 2, a rapid literature review was performed to identify established concepts for defining a research question in health research and data science as well as in other fields (refer to the Preparatory Research: Literature Search for the “Established Workflow” and an Example Scenario section). Expert inputs were gathered in a series of three 1.5-hour expert workshops. To foster a focused discussion in the workshops, participants were asked to complete preworkshop tasks. These inputs were summarized by JS and VvW and presented at the beginning of each workshop to discuss potential disagreements and allow participants to explain or comment on their and others’ inputs. The consensus and agreement of each objective were reached by an iterative, deliberative process. This included expert inputs before workshops, discussions during the workshop, and finally, expert feedback on and approval of the consolidated findings. These findings were synthesized, formulated, and shared by JS and VvW after each workshop. Furthermore, each participant was actively involved in the manuscript writing. These methods facilitated the systematic collection of input from participants in group and individual settings, enabling a comprehensive understanding of experts’ knowledge and consolidating diverse perspectives. Workshops were recorded after receiving consent from the team. Workshop minutes, including the results of the preworkshop tasks, were sent for approval to the expert group. When necessary, individual researchers were contacted after workshops for clarification on specific issues raised during the workshops. The documentation and reporting of the workshop and the Accurate Consensus Reporting Document (ACCORD) checklist [15] for the consensus methodology are available in Multimedia Appendices 1 and 2.

The first 2 steps were accomplished in workshops 1 and 2. Building on these results, a third workshop was dedicated to identifying the need for the adaptation of established research practices in the health field to streamline collaboration with data scientists and to better integrate and communicate the need for research principles and standards, including open science and reproducibility.

### Participants

The consensus meetings in the form of expert groups were led by JS and VvW, who led a previous project focusing on challenges and best practices of digital data, which inspired this study. Furthermore, JS is a scientific manager of the scientific community whose members were recruited for the consensus exercise. VvW’s expertise lies in epidemiology and digital health research, and JS’s expertise is mainly in digital ethics considering health research and health care. The workshop participants were recruited by JS and VvW among the diverse members of the Digital Society Initiative (DSI) Health Community at the University of Zurich. The members from the DSI were selected because it is a competence center for digital transformation that fosters interdisciplinary collaborations and projects studying the interplay and implications of digital transformations in society. Participants were included if they had experience with projects using digital data or planned to be involved in such projects. The workshop was promoted on the DSI website, through newsletters, and via word-of-mouth within the community. A total of 21 researchers from different disciplines and from all career stages participated in the workshops. This number of participants enabled to have an expert group with sufficient diversity to foster discussions and include insights from diverse disciplines. Of the 21 researchers, 13 (62%) represented health research, 3 (14%) represented data science, and 7 (33%) represented the social sciences and humanities.

### Preparatory Research: Literature Search for the “Established Workflow” and an Example Scenario

A rapid literature search was conducted to inform the planning of the workshops and to develop a project roadmap (by JS and VvW). To gather information on the established workflow for research question formulation (steps 1 and 2), we searched the literature for publications, reviews, and course guidelines written either in English or German in PubMed and Google Scholar databases (search terms are provided in Multimedia Appendix 1). The search was further complemented by retrieval of guidelines from universities in Switzerland, Germany, the United States, and the United Kingdom, for which we searched on selected university websites. In addition, coauthors contributed materials they were familiar with or had previously used for teaching or research purposes. On the basis of this literature, we proposed the initial model for the “established workflow” that combines existing well-established frameworks and practices. To guide the discussions of our workshops, we developed an example scenario of digital data reuse for health research, which was communicated to participants before the workshops (Multimedia Appendix 3).

### Ethical Considerations

The study followed the recommended procedures of the ethics committee of the Medical Faculty of the University of Zurich by completing the Data Protection/Ethics Self-Assessment Tool and received an exempt status. Participants were informed about the study’s scope and goals as well as the nature of their involvement. They provided consent before the workshop and were informed that they could withdraw from the study at any time without providing a reason. The participants did not receive any compensation for their participation. The only personal information collected for the study was sociodemographic data, which were anonymized.

## Results

### Establishing a Common Terminology

Anticipating that a lack of harmonization concerning terminologies and concepts may hinder an effective interdisciplinary workshop collaboration, we aimed to establish a shared understanding of common terminologies. To this end, the workshop leaders (JS and VvW) developed a glossary before the first workshop, which was discussed and further refined by collecting written feedback from the participants after workshop 1 (Table 1).

The workshop discussions concerning the glossary centered around discipline-specific interpretations of concepts such as “research task,” “research objectives,” “research aims,” and “research goals,” whose interpretations were dependent on the embedding in different research methodologies, such as qualitative or quantitative research approaches. A central discussion centered around the recognition of different “research tasks,” that is, high-level research aims from a methodological viewpoint, including, for example, exploration, confirmation, prediction, methods development, or theory development. For prediction and classification tasks, participants mentioned 2 subcategories of analyses, which are supervised learning methods that rely on labeled data and outcomes and include the broad class of (multivariable) regression models. By contrast, unsupervised methods (eg, neural networks) aim to find new data structures and features without the need for prior labeling and are often developed in a less linear, inductive manner.

**Table 1:** Common terminology for interdisciplinary research projects using digital data.

Terms	Definitions
Confirmatory research	Hypothesis-driven research, experimental research, or research aiming at testing and confirming a hypothesis in a broader context of a theory. This research is also referred to as hypothetico-deductive research in some disciplines.
Exploratory research	Data-driven research that aims at exploring new patterns and associations to formulate hypotheses.
Hypothesis	A tentative, hypothetical prediction of the nature and direction of relationships between sets of data, phrased as a declarative statement. It is an assumption about scientific laws, causation, or empirical regularities. A hypothesis should be testable or falsifiable. This refers to quantitative evidence-based health research.
Unstructured data	Raw data that are not in a predefined structure (eg, tables) or data that may be structured but still require substantial preprocessing or feature extraction (eg, continuous sensor data).
Principles and criteria of good research and research practice	A set of values and norms for good conduct of research, including validity, scientific integrity, objectivity, and ethical study conduct.
Research aim	The research aim is the overall, general, and long-term intention of a research project. The research aim describes the “what” of the research—where we aspire to be at the end.
Research design	Research design describes the general outline of data collection (eg, cross-sectional and longitudinal studies) and analytical methods (eg, randomization, observational, and with or without control group) to answer the RQ^a^. It describes the “how” of research.
Research objective	The specific goal linked to a RQ [16].
Research problem	The research problem describes the rationale for a study, for example, by highlighting the societal or medical needs. It describes the “why”—the specific needs a study wants to address.
RQ	A clear and concise question determining the research aim, objective, design, methodology, data collection, and analysis. The RQ narrows the aim and objective of the research. The process of defining a good RQ is dynamic and iterative. The RQ is refined through the different steps of the research cycle. We define the RQ in the context of quantitative evidence-based health research.
Research task	A research task describes a high-level classification of aims or tasks in research, including descriptive research, exploratory research, confirmatory research, prediction and classification, theory development, or methods development.
Reuse of digital data	The process of harvesting, transforming, and using structured or unstructured digital information that was initially generated for purposes other than research.
Theory or model	A systematic, structured explanation or representation of facts, phenomena, or processes that sets the ground for research design, formulation of hypotheses, and predictions.
Tools to specify RQ	Frameworks and tools that facilitate the development of specific aspects of defining the RQ or study design.
Types of RQ	The type of RQ determines the main approach for achieving the research aim. Usually, there is a difference between quantitative and qualitative RQs that reflect quantitative and qualitative approaches. Quantitative approaches use statistical and mathematical methods to address precise questions, typically using a deductive approach with a strong emphasis on the framework and structure. By contrast, qualitative approaches use, for example, open-ended responses, focus groups, and interview-based techniques and focus on individual experiences and singularities. It seeks to determine or discover a process or define experiences. RQs tend to be inductive, flexible, adaptable, and nondirectional [17].

^a^RQ: research question.

The group further discussed the central role of hypotheses and linear, highly structured research approaches in health research, for example, in confirmatory research tasks (confirming a hypothesis, eg, by use of randomized controlled experiments or trials) and, to some extent, research focusing on predictions tasks (developing prediction models or classifiers to predict future events or out-of-sample attributes). In health research, it is generally recommended that the development of prediction methods follows a protocol that includes careful selection of predictors and (external) validation of the final model [18]. At the same time, it was also pointed out that some quantitative research tasks, such as methods development (ie, the development and validation of data analysis methods) or exploratory research (ie, detection of patterns and associations to generate new hypotheses), as well as qualitative research, generally depend much less on the specification of hypotheses. Workshop participants with a qualitative background argued that hypotheses can be implicitly involved in the research project. In qualitative research, it is common for the research question to evolve due to the necessity to critically reflect and adjust the study focus in each research step. As a result, the overall research process in qualitative research and some quantitative tasks, such as methods development, can be more iterative and dialectical when compared to deductive or confirmatory health research.

These discussions led to a key insight that interdisciplinary collaborations may be streamlined through the identification and discussion of the most appropriate “research task” early on, which can help guide subsequent discussions about the research question and the role of hypotheses in a common direction.

### Summarizing Established Workflows for Research Question Formulation

**Figure 2:**
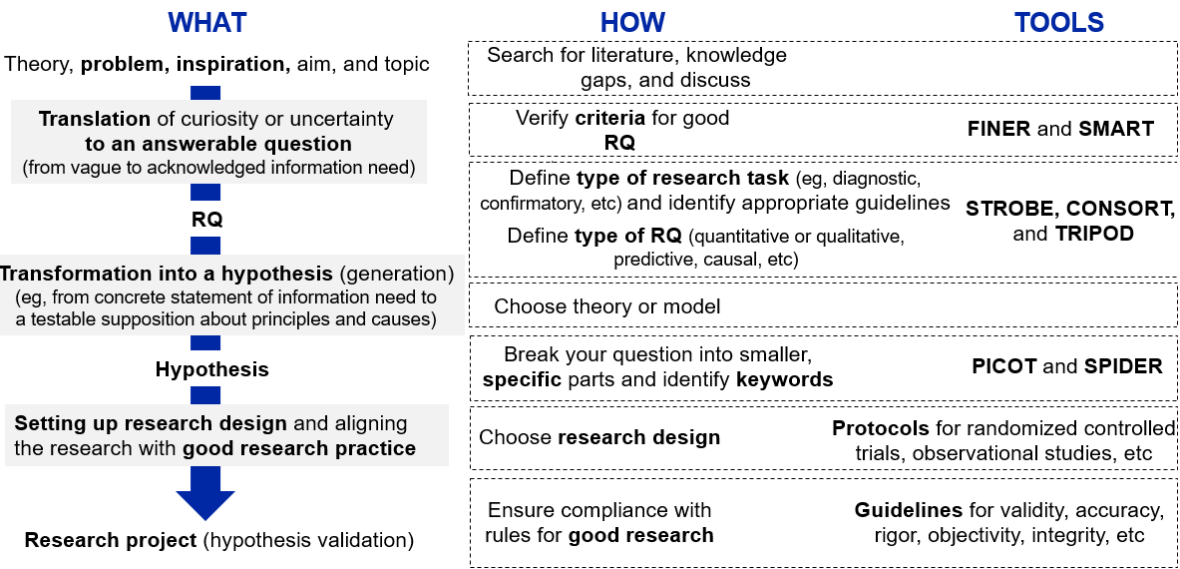
Workflow of the recommended practices for defining a good research question. CONSORT: Consolidated Standards of Reporting Trials; FINER: Feasible, Interesting, Novel, Ethical, and Relevant; PICOT: Population, Intervention, Comparison, Outcome, and Time; RQ: research question; SMART: Specific, Measurable, Achievable, Realistic, and Timely; SPIDER: Sample, Phenomenon of Interest, Design, Evaluation, Research Type; STROBE: Strengthening the Reporting of Observational Studies in Epidemiology; TRIPOD: Transparent Reporting of a Multivariable Prediction Model for Individual Prognosis or Diagnosis.

The first 2 workshops were dedicated to better understanding how different disciplines approach the initial steps of a research project, including research question definition and study design choices. Informed by our rapid literature review, Figure 2 illustrates a summary workflow for established research design practices in health research. The vertical axis of Figure 2 illustrates the recommended steps for defining a research question (the “what”), starting from finding inspiration to developing a hypothesis, designing an appropriate study, and validating the hypothesis. Aligned with these definition steps, Figure 2 displays established practices (the “how”) to execute the recommended steps. The third column references various frameworks and checklists aiding the implementation of each recommended step (the “tools”).

The workflow of established practices generally starts with identifying a meaningful problem or question to be addressed in a study. The inspiration often emerges from a real-world challenge or knowledge gaps, but it can also be derived from existing theories or be triggered by discussions among colleagues. Workshop members also mentioned the influential role of funding criteria (ie, to increase chances for funding success) or topic-specific funding calls. This inspiration, curiosity, or uncertainty then needs to be translated into an answerable question [19-21]. Although we found little guidance in the literature on how to operationalize this step, it is often recommended to check the research question against the FINER (feasible, interesting, novel, ethical, and relevant) and SMART (specific, measurable, achievable, realistic, and timely) quality attributes to ensure its suitability for testing in a research study [16,20,22,23].

The wording of the research question itself may already imply a specific research task (eg, exploratory, confirmatory, or qualitative research) [21]. We differentiate between research aim and research objective. Research aim is the overall goal of the research, whereas research objective is the specific goal linked to a research question [16]. Having clarity on the research task will also facilitate the identification of appropriate reporting guidelines, such as STROBE (for observational studies), CONSORT (Consolidated Standards of Reporting Trials; for randomized controlled studies), or TRIPOD (Transparent Reporting of a Multivariable Prediction Model for Individual Prognosis or Diagnosis; for the development of prediction models). These reporting guidelines primarily intend to guide the communication of study results but can also be useful in converting the research question into a study design.

Ultimately, decisions regarding the study design should be guided by the research question, while also considering practical limitations and available means and resources [23]. Frameworks such as PICOT (population, intervention, comparison, outcome, and time) and similar tools (eg, SPIDER [sample, phenomenon of interest, design, evaluation, research type] for qualitative research) [9,20,24] provide useful starting points for defining the study design. We include both the FINER and PICOT tools and their equivalents to ensure the best possible quality of the research question. Some research studies have shown that using only PICOT might be suboptimal [23]. The PICOT framework is frequently applied in health research, as PICOT is already defined above [21]. Further high-level study design decisions concern the study duration and measurement frequency (longitudinal vs cross-sectional studies), the allocation of study participants into comparator groups (randomization vs “as is” in observational research), as well as numerous practical aspects concerning study execution (eg, sample size and methods of data collection and analysis) [21,24]. Study design and study execution also need to adhere to the principles and criteria of good research and research practice to achieve valid, reliable, and accurate results [25]. Moreover, the research must comply with the standards of objectivity, reproducibility, and research integrity [26,27].

Overall, the workshop discussions confirmed that the workflow summary (Figure 2) represents a useful starting point for interdisciplinary collaborations to illustrate the established practices and to explore conceptual differences between health research, data science, and other scientific disciplines.

### Developing Recommendations for an Adapted Workflow

Building on the proposed example scenario for using digital data in health research (Multimedia Appendix 3) and the established workflow description from step 1, the participants then discussed 2 types of workflow adaptations to better reflect practices and approaches from data science (Figure 2). These included the following: (1) structural modifications by changing the sequence of workflow steps (ie, introducing additional steps that should become standard in a novel workflow—the “what”) and (2) the need for introducing additional contextual constraints or novel quality criteria (ie, modifications that do not change the workflow but may impact their execution—the “how”).

### Modifications to the “What”: The Steps in Research Questions Workflow

Overall, the workshop participants perceived that the general sequence of the established workflow (the “what”) still applies to studies using (structured and unstructured) digital data. Complementary steps with their potential pitfalls were proposed to better reflect the additional challenges of working with digital unstructured data (Multimedia Appendix 4.

First, for unstructured data, preprocessing and feature extraction should be allocated a distinct workflow step to emphasize the need for thorough consideration during study planning and execution, to ensure that the data are usable, credible, and useful for the research question at hand [28-31]. On one hand, the assessment of data quality and validity is more challenging. On the other hand, preprocessing and feature extraction through machine learning require additional assumptions and may lead to predictions and derived parameters with uncertain distributional characteristics (eg, normal ranges) or propagation of algorithmic errors and biases.

Second, the selection of appropriate analysis methods to address the research question as a new workflow step would underscore the importance of scientific rigor [1,31-38]. For example, deciding between pretrained deep learning models requires preliminary investigations about the model features and the training database, which goes beyond the choice of more standard statistical techniques (eg, regression models) [39,40].

Finally, the general importance of efforts to render science reproducible and transparent was identified as a new step in the workflow.

### Modifications of the “How” of the Research Question Workflow

The workshop group identified potential contextual and methodological changes to research practices (the “how”; Table 2). These proposed changes can be grouped into 3 clusters.

**Table 2:** Proposed changes to the “how” parts of the research question formulation workflow.

Change number	Contextual constraints and quality criteria	Description	Steps this is applicable to
I	Consider real-life incentives and constraints in defining research problems	The decision about RQ^a^ can be strongly influenced by other nonacademic factors such as the availability of funding or data.	RQ
II	Acknowledge feasibility and resource constraints	The choice of research design and data analysis tools involves costs that must be considered, particularly to ensure compliance with scientific integrity.	Research design
III	Declare limitations in RQ scope	Each RQ has limitations; it is important to define what RQ can and cannot answer.	RQ
IV	Allow for and document iterations in RQ development and analysis	Proper documentation is important for ensuring transparency and helps with evaluating and tracking the decisions regarding the iterations in RQ.	RQ
V	Acknowledge and respond to the increasing need for interdisciplinary expertise	For the feasibility of the RQ, it is important to consider the needed expertise and skills. This becomes particularly important in research involving digital unstructured data as it requires an interdisciplinary set of skills.	All steps
VI	Enhance reproducibility	Reproducibility in data science means obtaining consistent results using the same input data and methods. On a higher level, reproducibility in science also refers to the ability to duplicate findings if the same methods are used [41]. Reproducibility in science also refers to the concept of making data; computational steps, methods, and codes; and conditions of analysis transparent and available, so that others can verify the findings.	All steps
VII	Enhance replicability	Replicability refers to applying the same methods from a different study on different data. Observed differences in findings should be explicable by data-specific differences between studies.	All steps
VIII	Enhance robustness	Robustness refers to analyses that apply the same database but use different methods. Observed differences in findings should be explicable by method-specific differences between studies. Within the same study, robustness is often evaluated by sensitivity analyses that use the same data but vary methods (eg, by applying different model parameters).	All steps
IX	Critically assess generalizability	Generalizability means that the study results or outcomes are also applicable in other study settings and samples.	All steps

^a^RQ: research question.

The first cluster includes the acknowledgment of what we have labeled as real-life constraints (change numbers I and II) and limitations in the scope of research questions (change number III). Appropriately addressing such real-life constraints can be fostered by greater transparency and experience exchange.

The second cluster of proposed contextual changes pertains to enabling more interdisciplinary and iterative workflows (change numbers IV and V). Reasons for iterative approaches include more complex choices of analysis methods, the need for verifying the validity and robustness of model results, or the need to manually search for the best model parametrization. These challenges also require a greater emphasis on interdisciplinary collaborations that combine subject-domain knowledge and data science expertise.

The final cluster reflects the need for strengthening research quality criteria to foster open science, better reproducibility, and greater transparency (change numbers VI-IX). As analytical methods and databases become more complex, there is also an increasing need for transparency; adequate documentation; as well as publicly available analysis protocols, software codes, data, and analysis files. Studies should critically examine their findings under changing data or method combinations, thus exploring reproducibility, robustness, replicability, or generalizability (the 3RG criteria) and enhancing the overall quality of research. An important means to achieve these goals are open science and Findable, Accessible, Interoperable, and Reusable (FAIR) data principles [42].

### Recommendations Toward a Pragmatic Approach of Teaching and Conducting Research Question Formulation

The workshop discussions produced a set of specific recommendations to promote approaches for defining good research questions for reusing digital data. These recommendations are also well suited for educational use, helping to navigate the challenges of interdisciplinary collaboration and to foster interdisciplinary skills.

#### Iterative Research Question Formulation

As a principle, data collection, preprocessing, and analysis methods should follow the research question; researchers should not lose sight of the research aim, objective, and question. Defining a good research question is a fundamental and universal first step of science, which ideally should not be preceded by the choice of data or methods. However, the linear process of defining a research question common to health research may need several iterations to ensure that the complexity and feasibility of reusing and integrating digital data are accounted for.

The lecture instructors, supervisors, and principal investigators of interdisciplinary projects can apply this recommendation by emphasizing the importance of research question formulation in interdisciplinary projects. Furthermore, they can facilitate a discussion or create exercises for students to practice how the linear process of research question definition changes into a more iterative process when collaborating with other disciplines.

#### Reconciling Linear And Iterative Approaches: Continuum of Research Tasks

To reconcile the apparent conceptual differences between health research and data science approach research projects, we propose to reframe the scientific process as a continuum of knowledge accumulation over the course of multiple studies. Such a continuum can consist of several different research tasks (projects) combining deductive and inductive research approaches. Not all research tasks will involve explicit research questions or hypotheses. However, systematic reflections on how study results can inform new hypotheses and research questions and how they could be tested in future studies could become an integral part of a study, for example, as a last step in exploratory analyses.

Lecture instructors, supervisors, and principal investigators of interdisciplinary projects can use this recommendation to emphasize the continuum of research tasks. They can create exercises consisting of different research tasks where students practice combining deductive and inductive approaches in research design. These exercises can guide students to recognize that research is not always a straightforward process of hypothesis testing but may involve exploratory tasks that inform future studies. Instructors can also encourage students to reflect systematically on their research results, guiding them to think about how current findings can shape future hypotheses and research directions. This reflection can be incorporated into project work, where students work on iterative research tasks, examining how knowledge accumulates across studies and how inductive and deductive methods interact throughout this process. This practice prepares students to handle the nonlinear nature of interdisciplinary research, especially when bridging health research and data science.

#### Research Quality Criteria

The complexities involved in digital data preprocessing and analysis require careful design decisions and thorough reporting to ensure adherence to research quality standards. The reuse of existing, digital unstructured data and the need for extensive preprocessing may obfuscate or compound issues of external and internal validity [14]. Moreover, the use of machine learning techniques such as deep neural networks may generate “unexplainable” predictions or classifications that challenge the transparency and open science paradigms. The verification of “whether the data measure what they are supposed to measure (in the context of the research question)” [14] remains crucial and deserves appropriate attention, but it may become more difficult to achieve. We recommend that researchers systematically scrutinize interim results to ensure that they are “on the good track.” Such checks can, for example, include the replication of results from different studies. Furthermore, transparency in reporting and reproducibility are key to scientific rigor.

Lecture instructors and supervisors can emphasize the importance of maintaining research quality in interdisciplinary projects. They can design exercises where students practice making careful design decisions in their research projects, ensuring that issues of validity, transparency, and reproducibility are addressed throughout the process. One approach could be to guide students in developing protocols for systematic checks of their interim results. Instructors can also promote transparency by teaching students how to document their research processes thoroughly, facilitating reproducibility and open science principles. By applying these exercises, students learn to critically evaluate the quality of their research.

#### Take Active Measures to Foster Interdisciplinarity

We recommend reflecting these aspects appropriately in teaching and training of next-generation researchers as well as in establishing new interdisciplinary research groups or collaborations. Therefore, in teaching, it is important to also convey a realistic view of how research works in practice. Students should be sensitized to real-world challenges and the need for pragmatic decision-making, while still striving for the basic principles of “good research practices.” The literature review and our own experiences suggest that students are mostly taught the “ideal model,” and thus, they are often not well prepared for the realities of research. It seems preferable to discuss challenges openly and to expose students to ethical and practical dilemmas early on.

Lecture instructors and supervisors can sensitize students toward real-world challenges. They can prepare specific exercises where students can reflect on the problems that might arise from real-life constraints.

The added complexity and additional financial needs for education for interdisciplinary collaboration and open science should be acknowledged by funding agencies.

### Specific Tools to Inform the Teaching of Interdisciplinary Courses on Real-World Data Analyses

**Figure 3:**
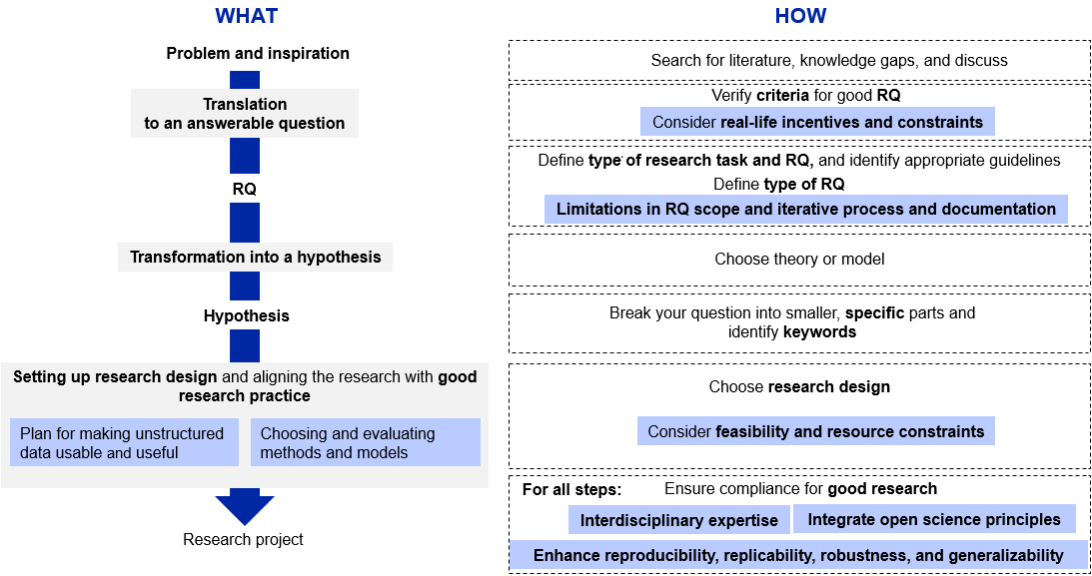
Adapted workflow. The blue-colored boxes contain the added aspects in the "what" and "how" categories.

Our study provides practical tools to guide the content and curricula of courses focused on interdisciplinary projects and collaborations. A more detailed description of the application of the study results to teaching is provided in Multimedia Appendix 5. The structure of our workshops (Figure 1) and the results of each workshop can be directly translated into the tools focusing on both dimensions of *content and curriculum* as well as *methods and teaching styles*. In terms of *content and curriculum*, the glossary with key concepts and terminology can be used to introduce students to interdisciplinary work. The workflow (Figure 2) combined with the glossary can serve as an interdiction to research practices for students with a different background, for example, humanities. Finally, our adapted workflow (Figure 3) sensitizes students for additional topics of transparency, FAIR data, reproducibility, and open science. Regarding *methods and teaching styles*, the sequence of workshops (Figure 1) and their results as outlined in the *content and curriculum section* can be directly translated into teaching phases, which build on top of each other. As illustrated by the example described in Multimedia Appendix 5, the structure of 3 teaching phases is useful and effective for teaching interdisciplinary research collaborations. A key insight from our workshop (modifications to the “how”—cluster 1) consisted of the need to acknowledge and address real-world challenges in study planning and execution. In our experience, case studies and illustrations of the scientific process of real-world examples are greatly appreciated by students.

## Discussion

### Principal Findings

This study examined how interdisciplinary research collaborations between health research and data science can be streamlined by creating a shared conceptual understanding of terminologies and best practice workflows and by acknowledging or merging approaches from other disciplines. In a series of interactive workshops, our interdisciplinary group of coauthors concluded that the workflow of established practices for formulating a research question, generating hypotheses, and defining research designs remain valid. We argue that the reuse of digital data does not substantially change scientific activity, particularly the fundamental step of defining a good research question [1,43]. Achieving clarity on the research question benefits data analysis and interpretation by providing structure and informing the study design workflow. Moreover, a shared understanding of the research question and study workflow facilitates the inclusion of diverse domain knowledge to ensure research quality and result quality [6,14,44]. In line with this, the group noted general tendencies in research toward more open, transparent, and reproducible research, which are influenced by recommended data science practices. Along those lines, health research should increasingly foster good scientific practices that help to align the reuse of digital data with principles of reproducibility, robustness, generalizability, validity [1,32], transparency, and open science.

Our set of tools and recommendations can also be integrated into medical education by providing academic educators with a structured approach to teaching research question formulation in the context of using digital data in health research. By emphasizing the importance of both hypothesis-driven and data-driven research methods, educators can guide researchers in navigating the interdisciplinary challenges of health research and data science. The importance of creating a common terminology and discussion about scientific principles can further increase awareness about the challenges of interdisciplinary collaboration between health researchers and data scientists. The proposed workflow and recommendations equip researchers with the tools to address the challenges of research question definition for interdisciplinary projects. The clear and practical steps provided by the workflow ensure that students not only grasp theoretical concepts but also apply them effectively in real-world scenarios, preparing them for collaborative, data-driven environments in health care and research.

For implementation, the set of tools and recommendations could be integrated into medical curricula and PhD programs through dedicated courses, workshops, or modules focusing on research methods and interdisciplinary collaboration for young researchers. Medical educators can adopt these recommendations to structure class discussions, assignments, and group projects, ensuring that students are exposed to both research approaches. In Multimedia Appendix 5, we provide an example of an interdisciplinary course implementing this set of tools. To evaluate the effectiveness of this implementation, a combination of qualitative and quantitative assessments can be used. Surveys and feedback from both students and educators can measure how well the workflow improves understanding and application of interdisciplinary research question formulation.

Our interdisciplinary effort recognized and discussed several potential obstacles toward bridging the approaches of established health research and data science. In the following sections, we repeat 4 key insights from our workshop interactions on how such obstacles can be overcome. First, we noted substantial differences in the use of terminologies across disciplines. For interdisciplinary collaborations, it is important to clarify key terms and concepts early on and to develop a shared understanding of the research aim and research question.

Second, in the early stages of the project, workshop participants expressed confusion about different types of analysis methods and their relationship with specific research tasks and high-level aims, such as prediction and classification, confirmatory research, or exploratory research. Agreeing on the high-level conceptual framework of “research tasks” helped structure the workshop discussions effectively. The discussion around the concepts of “research task” also fostered insights about commonalities and overlaps between concepts of data and health research. For example, many data science tasks can be classified as exploratory or prediction or classification tasks, which have conceptual counterparts in health research methodologies, each with corresponding reporting quality guidelines. Referring to specific research tasks rather than making global statements about data science or health research resonated well with the workshop participants and facilitated the discussions considerably.

Third, by introducing the concept of a “research task,” the group was also better able to examine the relationships among research aims, objectives, and tasks and how they are reflected in the workflow of established practices. Participants believed that exploratory or prediction or classification tasks, in particular, did not fit well into the workflow because such work is often not strictly hypothesis driven. However, 2 insights helped to align the workflow framework with the task concept: answering a research question may involve multiple research tasks in the same analysis, such as using prediction and classification tasks for data preprocessing, and later using these predictions in a confirmatory analysis, for example, as an exposure variable. Moreover, the scientific process can be viewed as a continuum of studies. From this perspective, the workflow of established practices can also be seen as a higher-level discovery cycle that spans across multiple studies. For example, an initial study may explore initial exploratory hypotheses or generate a first iteration of a prediction model, thus leading to new hypotheses. Indeed, exploratory and inductive methods can be useful to keep an open mind and become inspired by empirical data. In this way, the research tasks can be seen as a continuum—where data-driven research ends, hypothesis-driven research can start. Follow-up studies could then explore the hypotheses or validate the prediction model (whose structure can also be considered a hypothesis) in new data or in a confirmatory analysis. In combination, these multiple research tasks or study sequences are still likely to conform to the proposed workflow of recommended practices.

Finally, reusing unstructured and structured digital data brings new ethical challenges, such as privacy and consent issues, and problems with (public) trust and data diversity [45-49]. Traditional ethical assessments for data use in research and ethics review committees might not be well suited to address the challenges of digital data and might need adaptations [45,50]. Weighing the potential benefits and risks of using digital data becomes more complex. This problem is accentuated because the availability and production of digital data are often not based on a scientific decision, and rather, other factors such as political or social phenomena play a role [1]. While the need for novel ethical mechanisms to guide researchers is to be found in recently developed self-assessment tools for ethical data use [51,52], these new ethical mechanisms need further refinement to be widely adopted.

### Strengths and Limitations

The strength of the expert groups was that participants represented a diverse group in terms of disciplines and career stages. However, it is possible that not all potentially relevant viewpoints were represented. A further strength was that the inputs from experts were collected systematically via different channels (eg, discussions, preworkshop tasks, and commenting on documents) throughout the consensus process. This allowed to harmonize and synthesize knowledge and insights from diverse disciplines. Experts also had several opportunities to review discussion outcomes and final summaries through workshop protocol and involvement in manuscript writing.

There are limitations regarding our proposed workflow. First, it represents an idealized process for defining a good research question, which is often challenged by funding and resource constraints or established norms. Some parts of the workflow might not be explicitly applicable to all types of research. The example scenario used to develop the workflow was based on hypothesis-driven deductive research, which often uses relational and causal research questions. We did not explicitly include inductive, qualitative approaches in health research, but we see the deductive and inductive research on a spectrum [53]. This limitation does not prevent the overall concept of the workflow from being applied to other types of research, such as inductive, data-driven, or exploratory research.

Finally, although the literature review was conducted with great care and the expert group included several experienced researchers and faculty from different scientific disciplines, it was not possible to conduct a fully systematic search across all research disciplines due to resource constraints. Therefore, it is possible that some potentially relevant concepts and guidelines were not included.

### Conclusions

In an age of digital transformation, established scientific practices with a strong focus on formulating research question design remain relevant and useful for gaining clarity about research aims. We recommend initiating new collaborations in the health domain with a review of terminologies and concepts to avoid misconceptions and problems further downstream in the research process. Our terminology and workflow may serve as tools to be used in medical education to support young and established researchers in interdisciplinary health research projects. To this end, we found the concept of “research tasks” particularly useful to foster a shared understanding among our collaborators. In addition, we recommend adapting the way the established workflow is taught to prospective researchers in health research and other disciplines, incorporating concepts from open science, the 3RG criteria, and the “science as a continuum” paradigm. We also call for funding agencies and publishers to incentivize and acknowledge investments in defining good research questions for complex novel data and analysis methods.

## Appendix

Multimedia Appendix 1 Study and workshop protocols and preparatory tasks.

Multimedia Appendix 2 Accurate Consensus Reporting Document (ACCORD) checklist.

Multimedia Appendix 3 Example scenario of digital data reuse to structure workshop discussions.

Multimedia Appendix 4 Proposed modifications to the “what”: additional steps to the workflow.

Multimedia Appendix 5 Application of teaching tools: example from the course Interactive Data Science in Digital Health.

## Abbreviations

3RG: reproducibility, robustness, replicability, or generalizability
ACCORD: Accurate Consensus Reporting Document
CONSORT: Consolidated Standards of Reporting Trials
DSI: Digital Society Initiative
FAIR: findable, accessible, interoperable, and reusable
FINER: feasible, interesting, novel, ethical, and relevant
PICOT: population, intervention, comparison, outcome, and time
SMART: specific, measurable, achievable, realistic, and timely
SPIDER: sample, phenomenon of interest, design, evaluation, research type
STROBE: Strengthening the Reporting of Observational Studies in Epidemiology
TRIPOD: Transparent Reporting of a Multivariable Prediction Model for Individual Prognosis or Diagnosis

## Data Availability

All data are available in the manuscript and multimedia appendices.
