# Supplementary material for: Refining Established Practices for Research Question Definition to Foster Interdisciplinary Research Skills in a Digital Age: Consensus Study With Nominal Group Technique
Source: JMIR Med Educ. 2025 Jan 23;11:e56369. doi: 10.2196/56369 (PMC11803332; doi:10.2196/56369)
Supplement: Multimedia Appendix 1 [file mededu_v11i1e56369_app1.pdf]

Table of content

Study protocol (p. 2-9)

- Background & methods (p. 2-4)
- Milestones and project documentation (p. 5-6)
- Detailed project description (p. 7-9)
  - Step 1. Search syntax for a rapid literature review (p. 7)
  - Step. 2-3. Harmonization of terminology and established workflow (p. 7)
  - Step 4-6. Mapping challenges, developing and evaluating adaptions to the workflow (p. 8)
  - Project governance, execution and planned deliverables (p. 9)

Protocol of 1. Workshop (p. 10)
Protocol of 2. Workshop (p. 11-12)
Protocol of 3. Workshop (p. 13-14)

Homework for 1. Workshop (p. 15)
Homework for 2. Workshop (p. 16-)

Study protocol: How to define a good research question for studies involving unstructured digital data – a community project of the University of Zurich Digital Society Initiative

Jana Sedlakova, Viktor von Wyl

04.03.2022

Background

Digitization leads to a wealth of digital data that are potentially useful for secondary use in health research and care. Many of these data are, for example, a byproduct of administrative tasks such as keeping patient files or smartphone and social media usage. However, it is estimated that only a small fraction of this type of information is in a standardized ready-to-use format. The majority are unstructured data in different quantities, shapes and forms. Unstructured data are commonly defined as data that are not readily available in predefined structured formats such as tabular formats and/or require complex pre-processing and feature extraction to derive meaningful insights from the data. Typical examples are textual data (e.g., from electronic patient records), digital sensor data (e.g., from wearable sensors) or images (e.g., from CT or MRI).

Unstructured digital data can potentially be put to productive use in research and healthcare contexts, for example in a stand-alone analysis. Another possible use is the combination of unstructured data in the context of research studies that purposefully collect structured data. For example, longitudinal laboratory, clinical, or survey assessment may be complemented by additional unstructured data such as patient notes or smartphone information (e.g., mobility data or social media data) to glean further insights into the patient's lifestyle and behavior and consequently contribute to a better understanding of health risks or diseases. This process is also called "data enrichment", which is formally defined as a systematic process to complement collected data with additional relevant data from additional sources. In the context of this article, we will refer to "unstructured data enrichment" (UDE) as the process of complementing a structured database with unstructured information.

The main rationale for unstructured data enrichment is the augmentation of standardized data with, for example, real-time measurements and monitoring data in a natural living environment for diagnosis, prediction and prevention. These advantages potentially allow studying population groups that are under-researched or assessing behavior in long-term periods and outside of clinical settings. Including novel, more granular data can lead to better, more informative studies and a deeper understanding of participants' daily life contexts - thus enabling to gather study outcomes of greater relevance to the study participants. Ultimately, the results might foster personalized and more effective treatment.

However, unstructured data enrichment is associated with non-negligible challenges. Unstructured data often need complex preprocessing and preparation for meaningful feature extraction in a reproducible, robust manner when the choice of methods and techniques is not always straightforward. Other frequent challenges concern data quality characteristics, including data completeness, or a lack of standardization concerning the file formats or use of common syntax.

Further important challenges pertain to the often unclear suitability of unstructured data to address concrete research questions, as well as ethical and legal issues, such as an increasing danger of subject re-identification by use of unstructured data (e.g., from social media). In a recent literature review and through a survey among researchers at the University of Zurich, several such challenges were identified and summarized.

Moreover, there is a lack of high-level guidance for applied researchers on how to plan and approach unstructured data enrichment endeavors. For health research, several frameworks to aid research question development exist. Examples include the PICOT framework for research design specification or the FINER or SMART criteria to validate research questions. However, these existing concepts and frameworks need to be adjusted to the new universe of data at hand to reflect issues such as standardization problems, and the problem of alignment with a good hypothesis. Such challenges were recently compiled and summarized on the basis of a systematic literature and a survey among researchers who are affiliated with the University of Zurich Digital Society Initiative.

With this project, we aim to develop user-friendly guidance on how to develop a good research question when working with unstructured secondary, digital data (that is, data that were not specifically collected for a research purpose). We will focus on use cases that involve unstructured data enrichment but expect the newly developed guidance to be of relevance for any project that involves the secondary use of digital data.

Methods

General Approach

Through a series of literature screenings, community workshops, and consultation rounds we aim to develop a document that provides guidance on how to develop a good research question for studies involving unstructured digital data.

Although the majority of people in the working group (see below) have a background in health research, we aim to achieve a broad diversity of viewpoints and disciplines, which will include a broad search strategy for existing frameworks and consultation of experts from different research disciplines.

The development will follow a series of 5 interlinked, high-level steps. Specifically, we will

1) create an overview of existing frameworks for research question definition
2) develop a harmonized terminology for theresearch question definition
3) propose a workflow combining existing (published) frameworks for how to define good research questions (e.g. PICOT, FINER, SMART, etc)
4) identify gaps in the proposed workflow by mapping challenges for working with unstructured digital data
5) develop a proposal for a modified research question framework in order to fill the gaps identified in the step 3, and
6) evaluate the proposed framework in a structured community process (through qualitative interviews and online surveys)

These steps will be operationalized as a preparation phase (including initial research and discussion of frameworks and terminologies in a core team, a series of three workshops for framework development involving working group members recruited from the UZH-DSI community, as well as an

extended consultation and consolidation phase with broader involvement of researchers. Milestones and deliverables are listed in Table 1.

Table 1: Expected milestones and project documentation

Milestones / Deliverables	Project Documentation	Date accomplished
**Preparation phase**<br>● Definition of general aims and milestones as part of a grant application to the digital society initiative.<br>● Presentation of aims, milestones and general procedure in an online workshop.<br>● Broad information about the DSI health community via a roadmap document, an invitation to participate	DSI Health Community Proposal<br><br>Roadmap document and Email-Invitation for participation<br><br>Project Presentation (Kick-off)	March 2022
**Workshop 1: Overview of existing frameworks and clarification of terminologies**<br>● Identification of existing frameworks for research, both from literature search and through discussions in interactive, interdisciplinary online workshops<br>● Clarification of terminology in interactive, interdisciplinary online workshops<br>● Development of a high-level concept and workflow for research question definition, review and revision of workflow	Invitation to first workshop (Email and description of homework task)<br><br>Presentation at workshop<br><br>Milestones: workshop protocol, revised workflow	March 2022
**Workshop 2: Definition of concept and checklist for the definition of good research questions when using unstructured data**<br>● Identification of gaps and need for further specifications in the workflow<br>● Development of an adapted checklist/workflow proposal for research question development<br>● First-round review, discussion, and consolidation during the workshop<br>● Second-round review within the larger DSI health community in writing.	Workshop protocol, slides<br><br>Documentation of received feedback and implemented changes, version controlled documents	June 2022
**Workshop 3: Extended review/consultation and consolidation of augmented workflow**<br>● Before theworkshop: Further one-to-one exchanges/interviews with critical review by select experts	Workshop protocol, slides	November 2022

• First round consolidation of comments and document revision<br>• During theworkshop: discussion of remaining discrepancies, getting documents ready for broader consultation	Documentation of received feedback and implemented changes, version-controlled documents	
**Consultation phase**<br>Publication of draft document (white paper), invitation to provide comments, dissemination activities (e.g. online seminar). If needed: further expert interviews<br><br>Publication of complete white paper (version 1)<br>Manuscript for peer-reviewed publication	Documentation of received feedback and implemented changes, version-controlled documents	April 2023

Detailed project description

Step 1: Search for existing frameworks and clarification/harmonization of terminology

The literature will be searched for publications, reviews and university guidelines describing methods for research question development. The databases PubMed and Google Scholar will be searched using the following search string:

The following search strings and keywords will be used: (“research question”) AND (“defin*” OR “formulat*” OR “specif*” OR “develop*” OR “method” OR “framework”)

In addition, information about salient literature and frameworks will be elicited from the working group.

Step 2: Harmonization of terminology for research question definition

Based on a preliminary screening of the found literature, important keywords and terminologies are extracted, as well as definitions. Following this initial screening, a table of relevant keywords and definitions is compiled. This keyword table is reviewed and discussed during an online workshop. Subsequently, the table will be revised, complemented and shared for another (written) round of review.

Step 3: Development of a workflow proposal for the definition of good research questions

Based on our research into existing frameworks and current gaps, we will propose a workflow that integrates salient concepts and ideas from the literature. The role of this high-level workflow is to summarize and integrate different existing concepts. A preliminary version has already been developed based on preliminary literature searches during the preparation phase for this project (see below). This workflow will be further discussed and reviewed over the course of different workshops. The workflow will be accompanied by a commentary document, which describes different steps, includes key references, and provides examples.

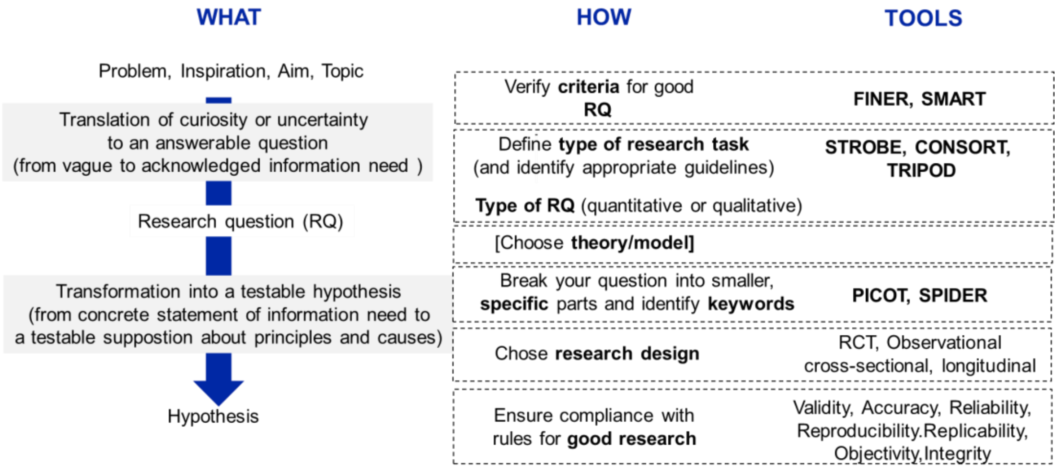

Figure 1: draft workflow as to be proposed in the first workshop

Step 4: Mapping of described challenges of using unstructured research data with existing frameworks to identify gaps

In previous projects of the core working group, a literature review was performed to compile challenges for researchers when working with unstructured electronic data (preprint publication forthcoming). Reported challenges were very broad, ranging from technical and infrastructure issues to a need for specific education. Furthermore, a survey among the research staff of the University of Zurich collected further input on specific challenges people have faced when working with unstructured research data. Overall, 177 responses were collected (manuscript writing in progress).

Based on these previously compiled challenges, we will explore in a workshop discussion whether and how these may relate to research question design and the proposed workflow (step 3) in particular. For example, reproducibility is a relatively recent topic, which ranks prominently in the list of research challenges but is – to our knowledge – not specifically linked to existing criteria and frameworks for research question development.

Ultimately, this mapping exercise will enable us to identify gaps in the proposed workflow (based on existing frameworks), and also to point out challenges that may be anticipated or even remedied at the research question development stage.

Step 5: Development of an augmented workflow proposal geared towards creating good research questions for the secondary use of digital data

Through a workshop and expert consultations, extensions to the workflow proposal will be developed. The extension development will be informed by practical use cases and databases (from the DSI community, https://health.dsi.uzh.ch/projects/ and other suitable projects), which will help to examine and test different modification proposals. These practice-inspired extensions should account for identified gaps and described challenges for unstructured data enrichment. The augmented workflow will be documented and described in an accompanying document.

In terms of development process and decision-making, we aim for a consensus process. In case a consensus is not achieved, diverging opinions and concerns will be documented (and ultimately published). Also important, the extended workflow will enclose all possible scenarios. Therefore, we will carefully describe our assumptions, development use cases, and possible limitations.

Step 6: Evaluation and refinement of the framework proposal in a structured community process (through written feedback and qualitative interviews)

The augmented workflow and the documentation will be opened for public consultation and expert reviews. In order to facilitate the process, we plan to conduct an online seminar where the augmented workflow and the development process are described, followed by a discussion round. Review feedback will also be elicited in writing (e.g. comments on the workflow draft and documentation files), as well as in separate expert interviews. The feedback will also be documented and made available as part of the project documentation. Exact procedures will be determined over the project course.

Project governance and execution

The principal investigator and a research assistant/PhD student lead the project, develop the materials, and organize the workshops. They are supported by an executive committee, whose role is to overview the project development process (including preparation and execution) and to act as a sounding board. The Execute Board comprised of voluntary members of the health community was established. This Executive Board is diverse concerning background and research disciplines (epidemiology, philosophy, linguistics, psychology, dental medicine, human medicine, communication science, geography) and in terms of career stage (PhD, Postdocs, senior researchers, Assistant and Full Professors).

Furthermore, a working group including members of the Executive Board and further DSI community members (and possibly select experts from outside the University of Zurich) was established. This working group consisted of all persons who made contributions to at least one of the workshops and were invited to contribute to the manuscript writing and revision (and thus become manuscript co-authors).

A total of 21 researchers from different disciplines and from all career stages participated in the workshops. This number of participants enabled to have an expert group with sufficient diversity to foster discussions and include insights from diverse disciplines. 12 researchers were early career researchers – PhD students and postdoctoral researchers. Health research was represented by 13 researchers, data science by 3 researchers, and social sciences and humanities by 7 researchers.

The augmented workflow proposal and commentary will be made available for comments, and contributors will be mentioned in the acknowledgement section of the manuscript.

Planned deliverables

The main deliverable will be a white paper including the augmented workflow proposal for research question development in studies using unstructured digital data. This white paper will include definitions and comments on the workflow steps. It is further planned to publish the white paper in revised form in a peer reviewed journal.

Furthermore, all project documents (slides, invitations, workshop documents) will be made available as a process documentation.

In a follow-up project we plan to design materials for a Master/PhD-level short course on research question development.

DSI-Approach: How to Design A Good Research Question for Projects with Digital Unstructured Data

1. Workshop «Terminology and Frameworks»

Protocol

11.3.2022, 2 -4 pm

Participants: 17

Summary

The workshop consisted of three parts that were accompanied by a lively discussion.

In the beginning, the **definition of unstructured data** was discussed. Different terms were proposed and discussed: naturalistic data, raw data; primary and secondary data, and secondary use of data. It was also proposed to formulate more precisely what is meant by "pre-processing" as every data needs to be preprocessed.
Result: A footnote with further explanation and terms can be added. The discussion will be re-opened and feedback will be collected in a later stage of the project.

Afterwards, the collected answers for the homework were presented and discussed. Finally, the developed model of how to define a good research question was presented and discussed. The main discussed topics were:

- To differentiate between **hypothesis-driven approach** (research question as a result) and **data-driven approach**; or approaches where the research question has a different life cycle (e.g., policy making, social science)
- A broader definition of "a **testable hypothesis**" could be useful as not every research conducts experiments
- Some types of research focus more on the **aim of research** than on a hypothesis
- Integrate the problem of how to define the **knowledge gap** in the research which might be for many challenging
- To what extent is the research question intertwined with the choice of **research methods**?
- To differentiate between fields/research that focuses on **knowledge gain vs method development**

At the end of the workshop, the group agreed to provide feedback on the presented model.

The next workshop will focus on how to adapt the model to specific challenges of projects that integrate unstructured data.

2. Workshop «Workflow & Concepts»

Protocol

8.6.2022, 3 - 5 pm

Participants: 12

Summary

The workshop consisted of two parts that were accompanied by a lively discussion.

In **the first half** of the workshop, we looked back at the first workshop, in which the **"workflow of established practices**" was developed. We clarified some **issues** that remained open or came up in the aftermath of the workshop. The main issues were:

- Can the "workflow of established practices" be used **for all research types** (e.g., data-driven/exploratory research)?
  ➔ The model is generic and can be used for all research types even though the exemplary case is confirmatory research. We see the scientific process as an iterative process on a continuum. Where some research types end (e.g., exploratory research to find a new hypothesis), other research starts (confirmatory research that tests the hypothesis). This means that the workflow can be adjusted and partially applied, for example, for exploratory research.

- Is the workflow applicable only **to health research**?
  ➔ No, the workflow should be applicable across different disciplines.

- Do we always need a **hypothesis**?
  ➔ Yes, but we refer to "hypothesis" in a broad sense: a) a testable hypothesis (confirmatory research), b) a general hypothesis in the form of expectations, thesis statements or assumptions aiming at achieving the research goal

Further remarks linked with these three issues:

➔ In the manuscript, we will comment on the differences across research types in more detail. We also make explicit that the workflow represents an **ideal**. Hence, some issues are not explicitly included, e.g., funding schemes that might determine the process of how a research question is defined. These issues will be described in the manuscript.

➔ The **terminology** for the two most discussed research types (confirmatory and exploratory research) was discussed from the methodological perspective. Supervised models are typically used for confirmatory research and unsupervised models for exploratory research.

➔ It has to be tested if the workflow is also applicable for research that focuses on **prediction**s.

In **the second half** of the workshop, we discussed the adjustment of the **workflow for research with digital unstructured data.** First, we presented a new workflow (slide 18) with added feasibility and quality criteria as well as steps that are related to unstructured data (see the attached PowerPoint presentation). The main discussion points were:

- ➔ Should we consider the cost-benefit when unstructured data are used?
- ➔ How can we integrate into the model different methodological approaches for unstructured data, e.g., when unstructured data are transformed into structured data or when the unstructured data are preprocessed to increase their quality?
- ➔ What criteria can be used to evaluate the performance of tools for unstructured data processing?
- ➔ Do we need to add intermediate results in the workflow?
- ➔ How much in detail do we need to describe data pre-processing, analysis etc.?

The attached slides (esp. slide 17) from the workshop illustrate the possible extensions of the “workflow of established practices” that have been discussed in the workshop. The color coding shows whether the group felt that an additional point should be integrated into a revised workflow or be mentioned as a “boundary condition” and discussed in the accompanying paper.

Finally, we discussed different **concepts and criteria for scientific research**, such as the role of **transferability/generalizability, reproducibility, validity, open science or transparency**. Specifically, we discussed the need for transparency and being transparent about the parts of the project where transparency cannot be reached and why (e.g., describing proprietary algorithms).

Next steps

- We will **finalize the workflow** for research with unstructured data
- Writing a **manuscript** to describe the workflow and address all issues that were discussed in the workshops
- In the **next meeting** in October/November, we will discuss the manuscript

3. Workshop «Consolidation of suggested changes and recommendations»

Protocol

18.11.2022, 9 – 10:0 am
Participants: 11

Summary

As a preparation for the final workshop, the first version of the manuscript was sent to the participants beforehand. The workshop was structured around three main topics:

1. Presenting and discussing the suggested changes in the workflow of established practices and recommendations
2. Discussing the first version of the manuscript
3. Discussing the next steps

1. Presenting and discussing the suggested changes in the workflow of established practices and recommendations

The discussion started with a general remark. Participants agreed with the proposal to understand different research study types and science as a continuum. This is particularly the case considering differences between exploratory and confirmatory research. The consequence of this approach is that the developed model for defining research questions is, in principle, valid for all types of research. The scientific method of defining the research question does not need substantial revisions given the new challenges of unstructured data. It was suggested to include this approach already in the introduction section.

Afterwards, a few changes were suggested regarding the table 2 and 3. It was suggested to emphasize the usability of data (which is the first suggested change in Table 2) in the text. Furthermore, it was discussed that including the additional step of open science might be a good pragmatic strategy for increasing awareness and emphasizing the importance of open science that gains importance in the context of digital unstructured data. It was also suggested to better formulate how machine learning models can be described. Regarding table 3, it was suggested to transform the table into a figure. The reason was that there are overarching elements (reproducibility, robustness, etc.) that are important for all steps of defining a research question.

2. Discussing the first version of the manuscript

First, the target group of the manuscript was discussed. It was agreed that the target group of the manuscript is a general audience and students in health research who might work with unstructured data. The manuscript does not aim to reach experts in the field. The goal of the manuscript is not to present a new, detailed workflow or standards. Instead, the goal of the manuscript is to present the topic of defining a good research question for projects with unstructured data from an overarching

perspective and increase awareness and understanding among students and researchers who are not familiar with this topic.

Several general suggestions for the content were made:

- To add the value of unstructured data with specific examples in the introduction
- To work with the third “w” – “why” throughout the manuscript
- To consider adding a new paragraph of possible exemplary pitfalls to avoid; as warnings – can be included in the “what” table
- To consider adding the importance of reporting negative results
- To consider adding more skills that are needed to succeed at each step of the workflow

Suggestions for discussion:

- Funding perspective
- Science as a continuum – it also means that studies need more time and resources, the continuum is getting longer
- Impact on the “who” – PI, coordinator, ethics committee and other regulatory agencies – what are the implications for ethics assessment?
- Ethics – what are the ethical constraints that can impact the definition of RQ? The question of proportionality – the ethical risk of using unstructured data vs. the value of their use (digital exhaust)
- Open future vision – what it means for future research – questions of sustainability (always new models and technology), scientific responsibility/integrity

3. Discussing the next steps

Finally, the next steps were discussed. After implementing feedback from the final workshop, the revised manuscript will be shared with the working group. The working group will provide feedback and comments on the manuscript. The introduction and discussion parts need particular attention. It was also discussed inviting experts to comment on the manuscript. The final manuscript will be shared with the DSI Health community and submitted to a journal focusing either on medical education or digitalization in health research. Ideally, the manuscript will be finished by the end of the year 2022.

DSI-Approach: How to Define Good Research Questions for Projects with Unstructured Data

1. Workshop "Frameworks & Terminology"

Homework

As preparation for the first workshop, we would like you to reflect on a few questions about how you generate research questions in your area of research. Please send your answers in written form to Jana (jana.sedlakova@ibme.uzh.ch) by March 7th. They will help us evaluate the diversity of approaches in the generation of research questions in different fields, as well as the suitability of our proposed framework to guide research question development. We will discuss the findings of these questions and our proposed framework in the upcoming first workshop.
Please also be aware that we may publish your answers anonymously as part of the project documentation.

General remark: Although we are aware that finding and generating research questions should follow a structured process, we are also aware that sometimes this process is not as structured and may be driven by intuition or a gut feeling. We are interested in an honest assessment, but would also very much appreciate your recommendations of frameworks and concepts that are used in your research field for research question development.

Question 1: How do you choose your research problems (i.e., broad topics of interest for your research)? For example, is it driven by general interest, experience in your field of research, intrinsic curiosity, or other reasons (or combinations thereof)?

Question 2: How do you generate specific research questions (i.e., a concrete question that summarizes the problem; the research question does not necessarily have to be specific with regard to methodology)? Do you have a specific strategy that you follow?

Question 3: Which frameworks/tools do you currently use or have used in the past for defining and refining specific research questions? If possible, please provide links.

Digital Society Initiative **DSI-Health Community**

DSI-Approach: How to Define Good Research Questions for Projects with Digital Unstructured Data

2. Workshop "Workflow & Concepts"

Homework

As preparation for our second workshop, we ask you for your support in two tasks.

First, we are interested in your **final feedback** on the revised **model** "How to define a good research question in confirmatory studies" (p. 2) that we have developed in the first workshop. Please send us any ideas for revisions, clarifications, etc.

Furthermore, in order to get the discussion started for the second workshop on how our "model for confirmatory studies" could be extended, please take a look at tables 1 and 2 from our systematic review on digital unstructured data enrichment. (p. 3 -11, attached to this document). Next, please **reflect on** the following **questions:**

1) Are there other challenges when working with digital unstructured data that are relevant for study planning of digital unstructured data projects?

2) How and where can these challenges identified in 1) be integrated into the model "How to define a good research question for confirmatory studies"? In other words, how should the model be extended in order to cover digital unstructured analyses?

Please send your answers in written form to Jana (jana.sedlakova@ibme.uzh.ch) by June 3rd. Your answers will facilitate the discussion in the upcoming workshop and help us to refine the model from our first workshops. Please also be aware that we may publish your answers anonymously as part of the project documentation.

Finally, we want to invite you to give us your feedback on the systematic narrative review if you are interested in the topic and co-authorship.

1.1 Model "How to define a good research question in confirmatory studies"

Defining Research Question

– The process of turning curiosity or uncertainty into an answerable scientific question

A good research question:

– narrows the research aim and objective

– guides the choice of methodology, e.g., methods, sample, sample size, data collection instrument and data analysis techniques

Model "How to Define a Good Research Question"

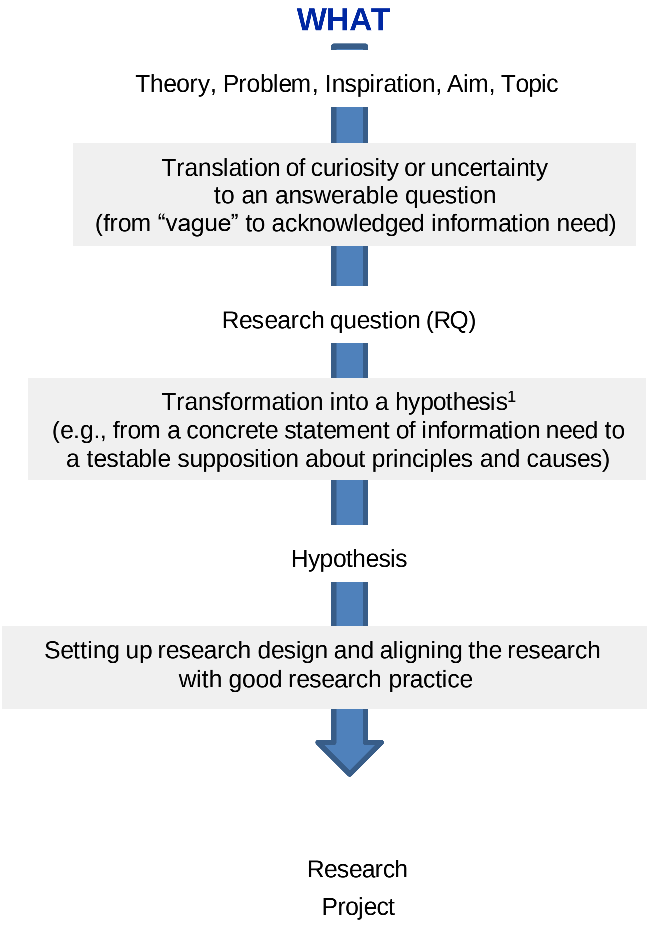

HOW	TOOLS
Search literature, look for knowledge gaps, discuss	
Verify **criteria** for good **RQ**	FINER, SMART
Define **type of research task**[1] (e.g., diagnostic, confirmatory, exploratory, predictive) and identify appropriate **guidelines**	STROBE, CONSORT, TRIPOD
**Type of RQ** (quantitative or qualitative, e.g., descriptive, causal, predictive, evaluative)	
[Choose **theory/model]**	
Break your question into smaller, **specific** parts and identify **keywords**	PICOT, SPIDER
Choose **research design**	RCT/experimental, Observational/correlational longitudinal/cross-sectional; prospective, retrospective
Ensure compliance with rules for **good research practice**	Validity, Accuracy, Reliability, Reproducibility, Risk-of-bias, etc.

[1] The role of hypothesis might differ depending on the research field - mainly considering the explanatory or data-driven research where an explicitly formulated testable hypothesis is not used. Thus, we refer to "hypothesis" in a broad sense that can include a) a testable hypothesis, b) general hypothesis in form of expectations, thesis statements or assumptions aiming at achieving the research goal. These assumptions might be crystallized in a specific form during the entire iterative research process. The formal and explicit step of formulating a testable hypothesis is mainly required for studies that address relational or causal research questions.

Table 1"Challenge areas" from systematic narrative review " Best practices for digital unstructured data enrichment in health research"

Challenge area	Definition/Description	Relevance
**1. Lack of meta-information for unstructured data**	All topics related to documentation of size, content, context, and format of unstructured data are included in this challenge area.	- To *scrutinize* and possibly *avoid biased* assumptions about the unstructured data (1).<br>- To provide *contextual information* for data interpretation (1, 12) (e.g., when and where data from wearable sensors were generated). The contextual information can enable a more *robust analysis* and *meaningful data interpretation* (12) which is important to *evaluate* the *accuracy* of the unstructured data *and* its *interpretability* (12).<br>- To facilitate the evaluation of the *quality and reliability* of the unstructured data (12).<br>- To facilitate the *interchangeability and reuse* of unstructured data (5, 15).<br>- To facilitate the process of *validation for regulatory acceptance* (e.g., in the context of digital health technologies (5)).
2. **Standardization Issues**	All challenges related to the conversion of unstructured data into a common standardized format that enables it to get shared, linked and used across different settings are included in this challenge area.	- To facilitate/foster effective data access, reuse of data for research projects and ultimately interoperability, interchangeability, and linkage of data (4, 5, 27, 28).<br>- To avoid duplication of research (5).<br>- To facilitate data interpretation and extraction of correct information (2, 3, 10, 13, 26).<br>- To enable data consistency (3).
**3. Data Quality and Bias in Data**	All topics concerning data accuracy, reliability, validity, and consistency are included in this challenge area.	- To increase/secure data accuracy, reliability, validity, and consistency.

		- To avoid a loss of efficiency in achieving study goals, errors in analysis.<br>- To facilitate interpretation of findings.
**4. Infrastructure**	All topics related to IT infrastructure that enable or facilitate data management, access, sharing, and processing are included in this challenge area.	- To facilitate access to data that researchers need for their research (2, 5).<br>- To avoid missed opportunities by lack of accessibility to relevant data sources.<br>- To reduce research costs (4, 10, 21).
**5. Finding suitable analysis tools, methods, and techniques**	All topics related to the methodological choices of how unstructured data are processed and analyzed are included in this challenge area.	- To facilitate the complex process of cleaning and analyses of large and complex datasets (1, 2, 3, 4, 6, 7, 13).<br>- To decrease risk for bias in research (7, 13).
**6. Alignment with a research design and/or research question**	All topics related to the broader theoretical issues of how the use and/or integration of unstructured data is linked with an appropriate research design and question is included in this challenge area.	- To ensure scientific rigor and validity (5).<br>- To determine the most suitable data analysis approach (12).
**7. Ethics & Legal Issues**	All topics emerging from ethical and legal concerns or risks either on the societal or individual level – such as privacy, confidentiality, safety, and discrimination (1, 2, 3, 5, 7, 9, 14, 17) – are included in this challenge area.	- Adherence to ethical and legal frameworks is a condition sine que non for research and requires no further justification.<br>- To facilitate a successful integration of unstructured data in health research as it can increase public trust and acceptance which might lead to increased availability of data sources.

Table 2 "Feasibility checklist" from systematic narrative review " Best practices for unstructured data enrichment in health research"

Key Issues	Comments
**Sufficient Metadata & Documentation for Unstructured Data**	Meta information can describe primary data and provide contextual information about data collection, pre-processing, or interpretation. Meta information is especially important for data that were collected for purposes other than research or data from wearables and other electronic devices.
Is meta information for the unstructured database available and where?	Meta information should be findable and well documented.
Can meta information offer sufficient contextual information for data interpretation?	Meta information should include:<br>Person: e.g., subject ID, medical history, or demographics<br>Context of collection: environment, study ID, or procedure description<br>Observations: e.g., technology-affiliated site location, technology type, or notes made by an observer (e.g., a clinician)<br>Time of data collection: e.g., time source, time zone, or medication schedule
**Standardization Options for Unstructured Data**	
Are data transformable into a standardized format?	A standardized format can be a tabular format. Many different standards for clinical data already exist. For example, for EHRs, the Fast Healthcare Interoperability Resources might be useful.[2]
Do data already contain standardized syntax/terminology or can such standards be applied?	Clinical information such as terminology and coding for diseases (e.g., ICD-10) might differ across databases. For data integration, sharing and reproducibility, standardized syntax and terminology are important.
Does the dataset contain standardized semantics/ontology, or can such standards be applied?	Standardized ontology describes logical relations between core concepts to structure the description of data and foster interchangeability and consistency of data. The problem arises when medical information has different expressions in data description.

[2] https://www.hl7.org/fhir/index.html

	For example, Systematized Nomenclature of Medicine—Clinical Terms (SNOMED CT) is a comprehensive medical terminology used for electronic health data.[3]
**Data Quality**	For observational studies, the checklist DAQCORD (daqcord.org) might be a useful starting point.
Can the consistency of data be secured? Are strategies/methods/steps available and included in the data management to secure consistency of data?	Consistency of data refers to the concept that the same data stored in separate places or separate time points still match.[4] For example, can be ensured that archived/backed-up/repository-deposited information can be kept up to date?
Is the dataset without a significant amount of missing data?	Due to selective reporting, EHRs may lack important data because clinicians did not deem them relevant, or patients did not want to share them.
Are strategies/methods available and/or defined for dealing with missing data?	Data may be missing for different reasons. They may be 'missing at random', they may be missing because the information was deemed irrelevant (e. g., not collected or not relevant for research question), because of branching or procedural logics (e.g., data are only collected under certain conditions). This knowledge also informs the feasibility of multiple imputation techniques, which assume at least some randomness (either systematic or non-systematic) in missing data.
**Data Validity**	
Are data only available for a limited population that might not be representative for an intended target population?	E.g., only a particular population group used the device collecting the data. Or physicians' notes are only available for a select subgroup (e.g., persons with a more severe clinical presentation). A sound understanding of the data generation process (possibly informed by meta information) is essential.
Are strategies implemented to prevent or minimize the risk that the data are affected by selection or information biases?	Biases can prevent that the measures or outcomes correspond to their true value. Epidemiological and medical research commonly distinguishes between three types of biases:[5] Selection bias occurs when the selection of study participants alters the exposure-outcome relationship (not to be confused with external

[3] https://www.snomed.org/

[4] https://en.wikipedia.org/wiki/Data_consistency

[5] Delgado-Rodríguez M, Llorca JBiasJournal of Epidemiology & Community Health 2004;58:635-641.

	validity/representativeness). Information bias occurs when the ways of how data is collected impair data accuracy. Confounding refers to an observed relationship between exposure and outcome, which is influenced by a third, unaccounted variable (e.g., "lung cancer is more prevalent among prevalent among persons who drink alcohol, but smoking is also associated with alcohol consumption"). Note that terminologies regarding biases may differ across scientific disciplines.
**Alignment with Research Question and Design**	
What is the purpose or motivation of enriching a dataset with unstructured data?	For example, unstructured data can provide additional insights into individuals' lived experiences or provide information in higher temporal resolution than standard data collection approaches (e.g., surveys).
Can the purpose of unstructured data be linked with a well-defined research question?	It is advisable to specify clearly defined and operationalized hypotheses before conceptualizing and conducting the study Considerations are needed whether unstructured data enrichment increases the chances for successfully testing of these pre-specified hypotheses.
Can the use of unstructured data be aligned with the planned research task (description, prediction, exploration, explanation, application)?	The purpose of unstructured data integration might differ depending on research tasks, e.g., description (such as describing a disease progression), prediction (predicting outcomes), exploration (to find new patterns or generate a new hypothesis), explanation (to establish causality) or application (such as the development of a practical tool for diagnosis).
Can the combined dataset lead to relevant, novel insights?	It should be considered what added value can be expected by the enrichment with unstructured data. Examples are: deepened qualitative or quantitative insights? More real-time time data? A stronger participant-centeredness?
**Infrastructure for Processing and Analysis**	
Does the research team have relevant skills/ or access to experts to approach for integration of unstructured data?	Interdisciplinary teams should include persons with strong (quantitative and/or qualitative) research methods skills as well as subject domain knowledge (e.g., specialists in a particular clinical area).

Can the interdisciplinary work be well established?	Define strategies to include persons with the necessary skills in the project teams, e.g., through existing networks, through consulting services (e.g., statisticians), referral by colleagues.
Can any duplication of research be excluded?	It is advisable to search for and summarize existing literature. It might be useful to check open data sources, platforms, database aggregators or searchable catalogues
**Availability of suitable analysis tools, methods and techniques**	
Are the appropriate methods, tools, and techniques available?	The integration of unstructured data with other data sources requires a set of different methods, tools and techniques from informatics, data science, coding, software development and others. The complexity of data opens many possibilities for analysis and statistical methods and their choice should be well justified.
Were the analysis methods, tools, and techniques chosen in a way that does not increase the risk of biases?	Analytical methods should ideally match the pre-specified study questions and hypotheses – not the other way around.
Can unstructured data be structured without significant loss of richness or other limitations? If no, will such limitations be reported/documented?	For example, qualitative information about patient experience from EHRs might get lost. Consider the integration of qualitative information as part of the analysis. Consider spot-checks and validation of quantitative findings using unstructured data (e.g., through random chart reviews).
**Expected quality of evidence of combined database**	
Can the methodology of hypothesis testing be well defined?	Given a set of pre-specified hypotheses: Are the data and planned methods suitable to detect the effect of interest (as indicated by, e.g., an a priori power analysis)?
Can the results be sufficiently validated to serve as research evidence?	Validation means the testing of (prediction) models and study findings in other, previously unused data. Validation pertains to testing whether the study findings can be applied to other similar individuals outside of the study and whether a statistical relationship, for example between cause and effect, can be generalized.
Can unstructured data be technically combined/merged with structured data?	What are the links between structured and unstructured data? Are there shared unique identifiers in both databases? Does the unstructured data need matching by specific time-points? Common challenges are that structured and unstructured data are not collected at synchronized time points and/or for all participants.

Can input from patient/population from whom the data was collected be meaningfully included in the study?	Including individuals' input about their experience, for example, with wearable sensors can provide important contextual information for ensuring quality and relevance of data and the study.
**Ethical and Legal Aspects**	
Have ethical and data security requirements been clarified and reviewed? If not, is it planned to contact relative authorities and regulators be contacted to clarify privacy and ethical requirements?	Many studies involving health data require approval by ethics committees. Moreover, it may be advisable to seek contact with data protection officers upfront to assess and identify potential data security and privacy risks
Have strategies for securing data privacy and security been clarified and are ready for implementation?	Linkage, processing, and analysis of unstructured and structured data require planning and consideration of the complete data life cycle. A data management plan should be put in place to outline rules and principles for handling data.
Can the data be fully anonymized or pseudonymized?	Deidentification efforts might not lead to full anonymization because an individual might be uniquely identified due to a specific piece or aggregates of information.
**Transparency, Reporting**	
Can all the steps of data management be well documented?	With the steps of data management, we mean data collection, data storage, data retrieval, data preprocessing, data analysis, data interpretation. Ideally, these considerations should be included in a data management plan and cover the full data life cycle.
Can be ensured that the documentation contain elements of established reliability, accuracy and validity of the studies?	Reliability refers to the stability of findings.<br>Accuracy is the proximity of measurement results to the "true" value.<br>Validity is the truthfulness of findings; the results of an experiment do measure the concept being tested.[6]

[6] Jonas Ranstam (2008) Methodological Note: Accuracy, precision, and validity, Acta Radiologica, 49:1, 105-106, DOI: 10.1080/02841850701772706.

rajković G. (2008) Measurement: Accuracy and Precision, Reliability and Validity. In: Kirch W. (eds) Encyclopedia of Public Health. Springer, Dordrecht. https://doi.org/10.1007/978-1-4020-5614-7_2081.

Is the analysis process being documented in a detailed fashion that allows sharing and replication?	This is a requirement for open science. A study protocol can be a good starting point for documentation. Moreover, all preprocessing and analysis steps should be programmed/coded and commented on.
Can the documentation support generalizability and replicability of studies?	Replicability is obtaining consistent results across studies aimed at answering the same scientific question.<br>Generalizability means that the study results or outcomes are applicable also in other study settings or samples.
Can limitations of data be reported?	For example, it should be reported whether unstructured data was only collected from a limited group of population or the collected textual data from social media was limited to long posts which might lead to a collection of data from a population group with specific characteristics.
Can the relevant technical steps of the data integration be reported?	This includes strategies, definitions and techniques for combining structured with unstructured data. For example, were data linked by person and for specific time points? What were assumptions and definitions used in the linkage process (e.g., was there a pre-specified time window within which two data points/assessments were considered as simultaneous)?
**Reproducibility of pre-processing, feature extraction and analysis, Open Science**	Reproducibility means obtaining consistent results using the same input data; computational steps, methods, and code; and conditions of analysis.<br>For reproducibility purposes and data sharing, FAIR principles might be particularly useful. FAIR[7] means “Findability, Accessibility, Interoperability, and Reuse of digital assets”.
Can it be ensured that the preprocessing steps reproducibly yield valid intermediary/analytical data?	See points above regarding documentation.
Can it be ensured that the feature extraction algorithms reproducibly yield data for meaningful statistical processing? Are extracted features sensitive to changes/adaptations in algorithms?	See points above regarding documentation. Documenting algorithms may be challenging when relying on proprietary software.

[7] https://www.go-fair.org/fair-principles/

Can raw data / intermediary data / analytical data be made openly available?	Whether and how data can be made available depends, for example, on data ownership, the availability of informed consents by participants, privacy risks, risks for re-identification.
Can raw data / intermediary data / analytical data be integrated into a well-designed open platform repository?	Many open data repositories with different requirements regarding data format or documentation exist. Many scientific journals demand a mandatory upload of certain data types into public repositories (e.g., for genetic sequence data).
