# Supplementary material for: Refining Established Practices for Research Question Definition to Foster Interdisciplinary Research Skills in a Digital Age: Consensus Study With Nominal Group Technique
Source: JMIR Med Educ. 2025 Jan 23;11:e56369. doi: 10.2196/56369 (PMC11803332; doi:10.2196/56369)
Supplement: Multimedia Appendix 3 [file mededu_v11i1e56369_app3.pdf]

1. Supplementary material: Example scenario of digital data re-use to structure workshop discussions

To guide the discussions of our workshops, we developed an example scenario of complex digital data re-use for health research, which was communicated to workshop participants prior to the meetings. In particular, we envisioned a scenario where health researchers aimed to enrich their current database (e.g., from repeated surveys) by re-using existing digital data (e.g., free text data from patient notes or social media) for a quantitative analysis. We further assumed that the analysis of the combined structured and often unstructured data involved complex pre-processing and data transformation steps, such as extracting sentiments or topics from the textual information. Due to the complexity and multi-modality of the combined (structures and unstructured) database, we assumed that more complex analytical methods, as well as an interdisciplinary data analysis approach was needed. For example, processing of text data increasingly relies on tools that build on pre-trained machine learning models of large text corpora (e.g., as provided on https://huggingface.co/), and the application of such models (for example to identify sentiment underlying text data), require an understanding of the foundations of machine learning, model fine-tuning, and interpretation of model outputs. Furthermore, machine learning-based analyses often do not have a clearly defined endpoint (i.e., a global maximum), and a key challenge is finding optimal model parameters in an iterative fashion. This scenario served as a starting point for a discussion on how such complex, iterative analyses can be aligned with traditional research paradigms that demand a clearly defined research question or hypothesis.
