# Supplementary material for: Refining Established Practices for Research Question Definition to Foster Interdisciplinary Research Skills in a Digital Age: Consensus Study With Nominal Group Technique
Source: JMIR Med Educ. 2025 Jan 23;11:e56369. doi: 10.2196/56369 (PMC11803332; doi:10.2196/56369)
Supplement: Multimedia Appendix 4 [file mededu_v11i1e56369_app4.pdf]

Supplementary Table 1. Proposed modifications to the "what": additional steps to the workflow

Additional step	Description	Rationale	Pitfalls
1. Making unstructured data usable, credible and useful	Data preprocessing: Data wrangling methods to transform the raw data into data that can be used in research. This includes restructuring, feature extraction, of harmonization of unstructured data, as well as data quality assessments. The data need to be credible – suitable and representative for analysis purposes- The data is useful if it is usable and credible (Kandel, Heer, Plaisant, Kennedy, Ham, et al. 2011).	Transparency and Reproducibility– preprocessing means making decisions about data quality and data structure. Preprocessing can data problems such as missing values or measurement error less transparent. Therefore, it is important to provide documentation and software code to make analytical decisions transparent and reproducible.<br><br>Awareness of potential information loss – preprocessing may involve simplifications and data reductions. If not documented and communicated appropriately, such preprocessing steps may blind analysts from the complexities and existing limitations in data.<br><br>Validity - The complexity of unstructured data brings a new dimension that needs proper validation efforts to ensure that it is measuring what it is supposed to measured (Hicks et al. 2019).	**Creditable data pitfall**: many real-world approaches fail to transform data in a way that it would be useful for the analysis task at hand. As a result, analysts run the risk of not systematically curating and extracting relevant signals, potentially leading to their oversight instead (Kandel, Heer, Plaisant, Kennedy, van Ham, et al. 2011).<br><br>**Pitfall of aggregation**. A key principle when it comes to big data challenges is to carve out relevant information and reduce unnecessary data. However, a considerable number of data science approaches runs the risk of overlooking important, interesting, and possibly unknown signals, e.g., by aggregating data through careless handling (Munzner 2014).<br><br>**Unusable data pitfall**: most data science workflows include a cascade of algorithmic models (e.g., machine learning models). Typically, every model requires data in a very precise syntactical form to even accept it as input. If the unstructured data is not faithfully transformed into this format, the data is just not usable for the workflow at hand (Fekete 2013).<br><br>**Data quality pitfall**: many domain experts consider their data to be in perfect quality, given the effort they have put into the data collection process. However, data quality is not binary, i.e., typically multiple quality issues need to be addressed and plausibility criteria be met. Typically, many data issues become apparent only after mitigating other masking effects. Particularly in non-iterative processes, data quality issues are often disregarded and not systematically curated and addressed. This results into the frequently cited "garbage in, garbage out problem" (Arbesser et al. 2017).
2. Choosing and evaluating methods and models for (unstructured) data analysis	Appropriate analysis methods capable of processing and analyzing available (unstructured) data while serving the research task	Evaluation and validation (Caliebe et al. 2019; Cerreta et al. 2020) - The use and performance of different tools and models in the process of data preparation, analysis and interpretation needs to be	**Pitfall when working with black box models**. Many scientific workflows include algorithmic models that are used as black boxes, i.e., algorithms running to completion in isolation with users only being aware of input and output, but not of the inner workings of the model. Downsides include difficulties in the parameterization, analysis, refinement, and

	and question need to be chosen.	regularly evaluated. The evaluation can be understood as systematic and numeric validation.<br>As an interim validation step, the replication of existing study results is recommended (Hicks et al. 2019).	optimization of black box algorithms. Mitigation strategies are carefully conducted and iterative design processes, testing and validation strategies also with different data conditions, to assess the efficacy of the models, and interactive white box solutions with having the human in the loop when/where need be (Sedlmair, Meyer, and Munzner 2012; Mühlbacher et al. 2014; Schreck et al. 2009).<br><br>**Pitfall of a lack of model explainability**: While proponents praise the AI as an ultimate solution for nearly any data-centric context, many machine learning models are complex and thus difficult to understand, interpret, and explain. This especially pertains to deep learning approaches. Especially in the healthcare domain this raises concerns about model trust, reliability, and explainability (Lipton 2017).<br><br>**Pitfall of a lack of evaluation**. The success of algorithmic data analysis processes is often assessed by quality metrics that need to be chosen with care. Using the well-known accuracy measure as an example, this measure only works meaningful for datasets with balanced class distributions. In contrast, research into visual quality assessment and human-centric validation have identified that, especially in highly domain-specific contexts, joint algorithmic and human judgment can be key to master evaluation and validation challenges (Munzner 2009; Bernard et al. 2011).
3. Fostering Open Science *<br><br>* no group consensus whether dedicated step is needed.	To ensure reproducibility of research, but also efficiency, it is important to share not only results, but all methodological steps. This can foster standardization issues that are one of the greatest challenges in the use of digital unstructured data.	Transparency gains on more importance because of the complexity of projects with digital unstructured data.<br>Transparency enables evaluation and reproducibility of research. It is not only controlling mechanism, but also driving force for further studies.<br>It is also important to maintain transparency even regarding projects components that might be unclear or uncontrolled,	

	Description of machine learning models used, if possible, incl. whether they were created by researchers or they were proprietary.<br><br>If possible, pre-registration and writing protocols as a controlling mechanism, but also to know what to expect from the study and results.	and to provide appropriate justifications.	
