# Supplementary material for: Refining Established Practices for Research Question Definition to Foster Interdisciplinary Research Skills in a Digital Age: Consensus Study With Nominal Group Technique
Source: JMIR Med Educ. 2025 Jan 23;11:e56369. doi: 10.2196/56369 (PMC11803332; doi:10.2196/56369)
Supplement: Multimedia Appendix 5 [file mededu_v11i1e56369_app5.docx]

**Application of Core Concepts in Interdisciplinary Teaching**

The focus of our study is on skill development to successfully navigate interdisciplinary collaborations and education in health-related research fields. The increasing digitalization and use of complex digital data have created a growing need for interdisciplinary collaborations, such as between health researchers and data scientists. However, methodological approaches and technical terminologies may vary across research disciplines, which poses challenges for productive and efficient interdisciplinary collaborations.

One major issue is the lack of structured teaching courses and guidance on how to effectively and constructively bridge different disciplines. Our study addresses this gap by offering a set of tools designed to facilitate the education and practice of transdisciplinary collaborations. These tools focus on two key dimensions: “Content & Curriculum” and “Methods & Teaching Style” and can be applied in various educational and research settings. Lecturers can use them to design interdisciplinary courses, supervisors can guide PhD and master's students in their interdisciplinary projects, and principal investigators can design and organize workshops to initiate and guide interdisciplinary projects. By implementing these tools, educators and researchers can create cohesive and productive educational resources for interdisciplinary collaborations. In the following, we offer our insights and a more detailed outline of how our study findings can inform both the Content and Methods dimensions, using an existing interdisciplinary course as an example.

**Section 1:** **Key insights and methods to inform teaching of interdisciplinary courses on real-world data analyses.**

Our study provides practical tools to inform the content and curricula of teaching focusing on interdisciplinary projects and collaborations. The structure of our workshops (Figure 1) and the results of each workshop can be directly translated into the tools focusing on Content and Curriculum as well as Methods and Teaching Styles.

1. Content and Curriculum
   1. Concept and Terminology (C & T). The Glossary (Table 1) can help to clarify key concepts and terms and serve as a starting point for discussions in interdisciplinary teams and introducing students to interdisciplinary work. It explains key concepts and terms from Health Research, Data Science, and Computer Science.
   2. Workflow and Practices (W&P). The workflow (Figure 2) illustrates a typical scientific sequence of steps in many research disciplines. It further lists tools and concepts that can inform specific steps in the research cycle. Combined with the glossary, the workflow can serve as an interdiction to research practices for students with a different background, e.g. in Humanities.
   3. Application and Collaboration (A & C). Our adapted workflow sensitizes students for additional topics of transparency, FAIR data, reproducibility, and open science. Despite the rising awareness of the importance of these issues, it is our impression that they seem to be treated as add-ons in teaching and not directly connected to the scientific process. Our adapted workflow calls for an integration of reproducibility and transparency into the standard process and specific recommendations are listed in Table 2.
2. Methods and Teaching Style
   1. The sequence of workshops (Figure 1) and their results as outlined in the Content and Curriculum section can be directly translated into teaching phases, which build on top of each other. Depending on the target group, course instructors, supervisors or principal investigators can use the following steps to guide and structure interdisciplinary courses and interdisciplinary projects. They can start with an introduction to high-level concepts for specific research disciplines and clarification of terminology [Concepts & Terminologies (**C&T**)]. Next, course instructors, supervisors or principal investigators can examine with their students or collaborators how different research disciplines approach the scientific process, and which tools or frameworks they commonly use to plan specific steps [Workflows & Practices (**W&P**)]. Finally, all stakeholders can identify strategies to overcome methodological differences and create synergies between approaches of different disciplines to foster transdisciplinary collaborations [Application & Collaboration (**A&C**)]. As illustrated by our example described in section 2, this structuring into the three teaching phases is also very useful and effective for teaching classes on interdisciplinary research collaborations.
   2. A key insight from our workshop (“Modifications to the “how” – cluster 1) consisted of the need to acknowledge and address real-world challenges in study planning and execution. Students are often taught very idealized principles and scientific workflows. However, experience shows that real-world study planning and execution is more iterative, messy, and underlying financial or other constraints. In our experience, students appreciate open accounts and real-world examples of how these dilemmas can be addressed (or not). In our experience, case studies and illustrations of the scientific process of real-world examples are greatly appreciated by students.

Our content and methodological recommendations for teaching and engaging in interdisciplinary collaboration are summarized in the section **“Recommendations towards a pragmatic approach of a research question formulation.” These recommendations touch both on content and style of teaching** offerings for interdisciplinary collaboration.

**Section 2: Teaching Example** **“Interactive Data Science in Digital Health”**

The “Interactive Data Science in Digital Health” course is co-organized by Profs. Jürgen Bernard (Computer Science) and Viktor von Wyl (Epidemiology & Digital Health) from the University of Zurich. The first iteration of this two-day block seminar took place in 2020. The course has been both an inspiration and a testing ground for some of the key insights developed in our manuscript. Below are a description and schedule of the course. In a separate paragraph we will outline how the findings from our research project have been integrated into the curriculum.

**Course Description**

Both data scientists and medical researchers such as epidemiologists conduct data-driven research to discover new knowledge and create evidence. Interestingly, the methodologies of both disciplines differ considerably, as you will learn in the seminar. At a glance, epidemiologists conduct carefully designed experiments to gather new data for downstream analysis, whereas data scientists exploit existing data, e.g., for visual data exploration purposes. Also, data visualization and interactive data analysis methods differ considerably in both domains.

How to evaluate new digital and mobile health applications? Which methods or study designs are most appropriate? What are legal and regulatory requirements? How can data quality problems be addressed? How to cope with the complexity of data? And when and how should users be involved in the development and evaluation process?

This two-day block course will address these and more questions from the viewpoints of clinical research methods on the one hand and interactive data science on the other hand. Together, we build bridges between a) experimental methods and concepts for evaluating medical health and b) statistical and machine learning tools and interactive data analysis methods. The focus will be on concepts, study planning, and the choice of analytic designs and methods.

The course will not be mathematical. Despite this, students should possess a basic understanding of data science tools such as statistical methods (e.g. linear regression). In the course, we plan for practical group exercise where students design a mobile health study.

In the weeks after the two-day block course, computer science students will conduct (continue) individual project work according to real-world application examples in digital health. The project results will be submitted to the teacher in form of a written document, according to a document structure pre-defined by the teacher.

**Target Audience**

MSc students or early-phase PhD students from Computer Science, Data Science, Health Sciences, and other quantitative disciplines.

**Learning Goals**

At the end of the seminar, students have gained a deeper and broader understanding of data-driven decision making in health applications.
In particular, participants should have an awareness for the critical points in study design and conduct, and for the data analysis workflow.
Given this knowledge, students will be able to critically appraise protocols and publications

Implicit Goal (communicated to students during first input lecture): We all will learn how to interact across disciplines, find a common language, appreciate other disciplines’ concepts and frameworks, and thus become better prepared for working and living in a highly digitalized and specialized world.

**Teaching Format**

Two-day block course, on-site instruction; lecture and group work

For informatics students: additional homework assignment (data analysis and report writing)

Two professors (Epidemiology/Digital Health and Computer Science/Data Visualization), 3 Teaching Assistants

**Teaching Schedule**

Phases: Concepts & Terminologies **(C&T);** Workflows & Practices **(W&P)**; Application & Collaboration **(A&C)**

| **Day 1** | **Starts** | **Ends** |
| --- | --- | --- |
| 0. Course Outline / Introductions / Housekeeping Remarks (15 Min) | 09:00 | 09:15 |
| 1. Research Methods for Digital and Mobile Health: Concepts and Applications **(C&T)** | 09:15 | 09:45 |
| Short Break (10 Min) | 09:45 | 09:55 |
| 2.How do Epidemiologists and Data Scientists Think? (60 Min) **(C&T)** | 09:55 | 10:55 |
| Practical 1 (incl. Short Break): Terminologies, Interdisciplinary Discussions (35 Min) **(C&T)** | 10:55 | 11:30 |
| Plenary Practical 1 (15 Min) | 11:30 | 11:45 |
| **Lunch Break** | **12:00** | **13:00** |
| 3a. Research Methods for Real World Analyses: Case Study, Basic Concepts (40 Min) **(W&P)** | 13:00 | 13:40 |
| 3b. Research Methods for Real World Analyses: Confounding (35 Min) **(W&P)** | 13:40 | 14:15 |
| Break (15 Min) | 14:15 | 14:30 |
| 3c. Research Methods for Real World Analyses: Randomization, Analysis Strategies (35 Min) **(W&P)** | 14:30 | 15:05 |
| Short Break (10 Min) | 15:05 | 15:15 |
| 3d. Research Methods for Real World Analyses: Concepts and Applications (35 Min) **(W&P)** | 15:15 | 15:50 |
| Roundup Day 1 (10 Min) | 15:50 | 16:00 |
|  |  |  |
| **Day 2** | **Starts** | **Ends** |
| 4a.Data scientists’ approaches to analyzing (non-experimental) data (45 Min) **(W&P)** | 09:00 | 09:45 |
| 4b.Data scientists’ approaches to analyzing (non-experimental) data (45 Min) **(W&P)** | 09:45 | 10:30 |
| Break (15 Min) | 10:30 | 10:45 |
| Practical 2: Vizualization Exercise – The not so good, the bad, and the ugly (30 Min) | 10:45 | 11:15 |
| Plenary Practical 2 (30 Min) **(W&P)** | 11:15 | 11:45 |
| **Lunch Break** | **11:45** | **12:30** |
| 5. Special: Invited lecture and interactive application example by a Digital Health Start-up (60 Min) **(A&C)** | 12:30 | 13:30 |
| Short Break (10 Min) | 13:30 | 13:40 |
| Continuation: Plenary Practical 2 (+15 Min) **(A&C)** | 13:40 | 14:05 |
| 6a. User Engagement (20min Participatory Design) | 14:05 | 14:30 |
| 6b. User Engagement (20min Think-Pair-Share) | 14:30 | 14:40 |
| Short Break (10 Min) | 14:40 | 15:40 |
| 7. Panel on Data, Users & Tasks. Example: Multiple Sclerosis Activity Patterns (60 Min) **(A&C)** | 15:40 | 15:50 |
| Short Break (10 Min) | 15:50 | 16:00 |
| Roundup Course (lessons learned, Feedbacks) (10 Min) **(A&C)** | 16:00 | 16:10 |
|  |  |  |
| **After the Course** |  |  |
| Homework Offering (for Computer Science Students Students) |  |  |

**Example Exercise for “Interactive Data Science in Digital Health”**

**Developing a common understanding of key concepts in epidemiology and data science**

Goal: to recognize and mitigate challenges in defining a common terminology across disciplines. **(C&T)**

Format: Guided group discussion (6-8 students, 1 teaching assistant)

Students should be able to recognize and discuss key concepts of epidemiology and relate them to similar concepts in their own discipline (if applicable).

Groups should quickly review the following concepts (using the lecture slides).

- Bias
- Generalizability
- Validity and Reliability of Measures
- Iterativity of data analysis
- Usable and Useful Data
- Evidence and Knowledge

As a group, discuss the following aspects:

1. Which terminologies are a) common in everyday language and b) in the context of your discipline. Are there conflicting definitions or ambiguities?

2. Do you have own experiences where interdisciplinary collaboration was hindered by different concepts and terminologies? How did you handle this situation? Or what would you recommend?

**Comment: Integration of key insights from our paper into the course**

Our course is designed to bring students from different disciplines together and let them experience the challenges and opportunities of transdisciplinary interaction and collaboration. Through input lectures and group exercises, they are exposed to concepts and terminologies they are – at least partially – not familiar with. Using plenary discussions and group sessions as instruments, the instructors (Professors, Teaching Assistants) try to create a dialectic dialogue between disciplines. When one instructor is presenting content, the others will add comments and share their experiences in working in interdisciplinary environments or how specific concepts translate to their field. This creates a lively classroom environment where students are encouraged to learn how other disciplines think. Students are also engaged in discussions and exercises to learn how to combine the research approaches from health research and data science. The course also reserves one slot for external speakers (for example, a patient with a chronic disease, start-up entrepreneurs in digital health) to share their views on communicating with people from different professions and backgrounds. This sensitize students towards real-life challenges and constraint that might come alongside interdisciplinary projects.

**This course integrates the major elements discussed in our research project**

1. Content and Curriculum
   1. Clarify key concepts and terms: Sessions 1 and 2 expose students to different ways of thinking in Health Science and Data Science. In a group exercise, students try to develop a common understanding of key concepts and terms. They are sensitized towards ambiguities in how terminologies are used in different settings and environments. They are also encouraged not to assume that everyone understands everything and plan accordingly.
   2. Typical scientific workflow. Sessions 3 and 4 confront students with established research practices in different fields. Examples and case studies from instructors’ research are used to illustrate research practices. While one instructor leads and presents, others comment and provide their view. This creates a dialogue and a lively classroom atmosphere where students are encouraged to contribute to the discussion and compare methods and practices from their field with another field.
   3. Our lecture weaves important concepts of transparency, FAIR data, reproducibility, and open science into the lecture to increase awareness of the concepts as well as introduce them directly into the scientific process. These concepts are particularly important in the context of interdisciplinary research when different methodologies are applied. Especially Session 7 aims to present a combined Data Science / Health Science approach on the basis of a joint research project of Profs. Bernard and von Wyl.
2. Methods and Teaching Style
   1. Three teaching phases. The three proposed phases of Concepts & Terminologies (C&T); Workflows & Practices (W&P); Application & Collaboration (A&C) can be used to structure the teaching scheduleas indicated by labels and color codes in the lecture schedule.
   2. Acknowledge and address real-world challenges. Our lecture relies on own research and case studies. The examples are designed to provide a realistic overview and insights into real-world data analysis challenges. For example, the first part of lecture 3 illustrates the full process of research planning, analysis, and planning using a project conducted by Prof. von Wyl. This case study includes in-depth explanations of analysis design decisions by running and commenting statistical analysis code in real-time. Moreover**,** students are also encouraged and have ample opportunities to share their own experiences and potential data analysis struggles with teaching assistants and instructors in a safe, constructive environment.
